# Educational attainment and diabetes risk: triangulation evidence from UK Biobank prospective cohort, NHANES 2011-2018, and cross-trait genomics analyses

**DOI:** 10.3389/fendo.2026.1867159

**Published:** 2026-06-26

**Authors:** Guannan Geng, Shizheng Qiu, Zhishuai Zhang, Xinru Liu, Xin Wang, Yang Hu, Hongyu Kuang, Jiahui Zhang

**Affiliations:** 1 Department of Endocrinology, The First Affiliated Hospital of Harbin Medical University, Harbin, China; 2 Center for Bioinformatics, Faculty of Computing, Harbin Institute of Technology, Harbin, China; 3 Key Laboratory of Biological Bigdata, Ministry of Education, Harbin Institute of Technology, Harbin, China; 4 Department of Stomatology, The Fourth Hospital of Harbin Medical University, Harbin, China; 5 Heilongjiang Provincial Key Laboratory of Hard Tissue Development and Regeneration, Harbin, China; 6 School of Stomatology, Harbin Medical University, Harbin, China

**Keywords:** body mass index, educational attainment, GWAS, mediation analysis, prospective cohort, type 1 diabetes, type 2 diabetes

## Abstract

**Background:**

Lower educational attainment is associated with obesity and type 2 diabetes (T2D), but prospective evidence, external validation, mediator patterns, and genetic triangulation have rarely been integrated.

**Methods:**

UK Biobank analyses included 501,932 participants at baseline and 474,659 participants without baseline diabetes prospectively. Logistic and Cox models estimated associations of higher educational attainment with prevalent and incident diabetes. T2D pathway analyses evaluated adiposity, health behaviors, and cardiometabolic biomarkers. NHANES 2011–2018 provided weighted cross-sectional validation. Public GWAS summary statistics were used for genetic correlation, Mendelian randomization (MR), tissue enrichment, candidate-gene analyses, shared-locus analyses, and S-PrediXcan transcriptome-wide association analyses (TWAS).

**Results:**

In fully adjusted UK Biobank models, higher educational attainment was associated with lower odds of prevalent T2D (OR 0.76, 95% CI 0.72-0.79) and lower risk of incident T2D (HR 0.70, 95% CI 0.68-0.72). Detailed qualification categories showed lower prevalent T2D odds for college/university degree versus no qualifications (OR 0.69, 95% CI 0.65-0.72). In NHANES, college graduation or above was associated with lower prevalent T2D odds (OR 0.69, 95% CI 0.61-0.78; n=20,502). Adiposity, smoking, alcohol use, lipids, blood pressure, and C-reactive protein attenuated the education-T2D association. Genetically predicted educational attainment was inversely associated with BMI and T2D, and BMI/T2D brain TWAS identified 19 shared multi-SNP gene signals, including NPC1, HSD17B12, MAP2K5, DHX36, BHMT, and LEPROT.

**Conclusions:**

Higher educational attainment was consistently associated with lower T2D risk across UK Biobank, NHANES, and genetic analyses. The results highlight modifiable metabolic and behavioral pathways relevant to T2D prevention.

## Introduction

Diabetes is a leading cause of premature morbidity, disability, and mortality worldwide. Although type 1 diabetes (T1D) and type 2 diabetes (T2D) differ substantially in pathophysiology, both impose major clinical and public health burdens. Educational attainment is increasingly recognized as a fundamental social determinant of health that may shape health literacy, employment opportunities, income, psychosocial stress, lifestyle behaviors, and access to healthcare (1–3). Observational studies have consistently linked lower educational attainment to a higher risk of obesity and T2D, yet the magnitude, independence, and mechanistic basis of these associations remain incompletely understood (1, 4).

Several pathways could explain why education relates to metabolic disease. Individuals with higher educational attainment are, on average, less likely to smoke, may differ in alcohol use patterns, may engage in more favorable health behaviors, and may have lower adiposity and better cardiometabolic profiles (5–8). Prior work has also suggested that obesity may mediate part of the association between education and T2D (1, 9–14). At the same time, educational attainment is correlated with cognitive performance and intelligence, making it difficult to determine which education-related phenotype is most relevant to metabolic risk (15, 16). Mendelian randomization (MR) studies have suggested that higher genetically predicted educational attainment may reduce T2D risk, potentially independently of cognitive performance (8).

Important gaps remain. First, although the association between education, obesity, and T2D has been reported previously, fewer studies have integrated large-scale prospective observational analyses, formal mediation analyses, external validation, and cross-trait genetic evidence within the same framework. Second, it remains unclear whether associations with T1D resemble those with T2D or are substantially weaker, particularly because adult-onset T1D in population cohorts may include latent autoimmune diabetes in adults or misclassified diabetes. Third, genetic studies have identified links between educational attainment, obesity, and T2D, but pathway-level interpretations remain indirect and require careful calibration.

To address these gaps, we used a triangulation framework combining observational analyses in UK Biobank, cross-sectional validation in NHANES, and genome-wide cross-trait genetic analyses. The study examined associations of educational attainment with prevalent and incident T1D and T2D, evaluated pathways involving adiposity, health behaviors, blood pressure, lipids, and inflammation, and integrated genetic correlation, MR, tissue enrichment, candidate-gene, shared-locus, and TWAS analyses. The main emphasis was the consistency and potential pathways of the education-T2D association, with T1D presented as a secondary diabetes-record outcome.

## Methods

### Study design and analytical framework

The study combined observational analyses in UK Biobank, external cross-sectional validation in NHANES, and genome-wide analyses based on publicly available GWAS summary statistics. The observational component quantified associations of educational attainment with prevalent and incident diabetes and evaluated potential mediating pathways. The genetic component assessed shared genetic architecture between education-related traits, obesity, and diabetes and prioritized candidate tissues, loci, and genes.

### UK Biobank population

UK Biobank is a large population-based prospective cohort that recruited more than 500,000 participants aged 40–69 years across the United Kingdom between 2006 and 2010 (17–19). At baseline, participants completed touchscreen questionnaires and interviews, underwent physical measurements, and provided biological samples. Long-term follow-up is available through linkage to hospital, death, cancer, and other health-related records (20).

Educational attainment was defined using UK Biobank field 6138. In the primary analysis, participants with a college or university degree or a higher qualification were classified as having higher educational attainment; all others were classified as not having higher educational attainment. Secondary analyses used detailed qualification categories derived from field 6138 with a highest-observed-qualification hierarchy: none of the above, CSEs, O levels/GCSEs, NVQ/HND/HNC, A levels/AS levels, other professional qualifications, and college/university degree. T1D and T2D were identified using UK Biobank first-occurrence fields 130706 and 130708, respectively. Recorded dates distinguished prevalent disease at baseline from incident disease during follow-up. Age at first occurrence, overlap between T1D and T2D records, and self-reported doctor-diagnosed diabetes were summarized to characterize diabetes-type classification. Prevalent T1D and prevalent T2D were defined as diagnoses occurring on or before the baseline assessment date. For prospective analyses, participants with diabetes at baseline were excluded. Incident T1D and incident T2D were defined as diagnoses occurring after baseline and before death or administrative censoring on September 1, 2025, whichever came first. Follow-up time was calculated from the baseline assessment date to the date of diagnosis or censoring.

### Covariates and candidate mediators

Demographic covariates included age, sex, and ethnicity (White versus other). Lifestyle covariates included current smoking, current alcohol use, and physical activity. Current alcohol use was defined from UK Biobank field 20117 as current drinking versus previous, never, or prefer-not-to-answer; alcohol intake frequency from field 1558 was summarized descriptively. Clinical and metabolic variables included BMI, waist circumference, systolic blood pressure, antihypertensive medication use, triglycerides, HDL cholesterol, and C-reactive protein. For incident T2D, candidate mediators included BMI, current smoking, current alcohol use, waist circumference, systolic blood pressure, triglycerides, HDL cholesterol, and C-reactive protein. For prevalent T2D, BMI, current smoking, current alcohol use, and waist circumference were additionally examined in mediation analyses.

### Statistical analyses in UK Biobank

For cross-sectional analyses, logistic regression estimated odds ratios (ORs) and 95% confidence intervals (CIs) for associations of higher educational attainment with prevalent T1D and prevalent T2D. For prospective analyses, Cox proportional hazards models estimated hazard ratios (HRs) and 95% CIs for incident T1D and incident T2D. Three nested models were fitted: Model 1 adjusted for age, sex, and ethnicity; Model 2 additionally adjusted for current smoking, current drinking, and physical activity; and Model 3 additionally adjusted for BMI. Missingness was summarized for core variables, and regression models used complete-case data for variables included in each model. Proportional hazards were evaluated using early and late follow-up analyses split at the median event time, with fully adjusted HRs compared across time windows.

Potential pathways linking educational attainment to T2D were evaluated using single-mediator analyses with bootstrap resampling. The indirect effect was approximated as the product of the education-to-mediator effect and the mediator-to-diabetes effect on the model link scale, and 95% CIs were obtained by bootstrap resampling. Because candidate mediators are correlated, joint mediator-adjusted models simultaneously included smoking, current drinking, BMI, waist circumference, systolic blood pressure, triglycerides, HDL cholesterol, and C-reactive protein. Stratified prospective analyses for incident T2D were conducted by age (<60 vs. >=60 years) and BMI (<30 vs. >=30 kg/m^2^).

### NHANES 2011–2018 validation

NHANES 2011–2018 data were used for external cross-sectional validation. Four two-year cycles (2011-2012, 2013-2014, 2015-2016, and 2017-2018) were merged using demographic, diabetes questionnaire, body-measure, and smoking questionnaire files. Analyses included adults aged >=20 years with diabetes questionnaire data. Higher educational attainment was defined as college graduate or above (DMDEDUC2 = 5). Prevalent T2D was defined as self-reported doctor-diagnosed diabetes with age at diagnosis >=30 years; participants reporting no diabetes were classified as controls. Weighted baseline characteristics were summarized using combined MEC weights. Weighted logistic regression estimated the association between higher educational attainment and prevalent T2D, adjusted for age, sex, race/ethnicity, current smoking, and BMI.

### GWAS data sources for genetic analyses

We obtained genome-wide association study (GWAS) for educational attainment and cognitive performance from Social Science Genetic Association Consortium (SSGAC), and GWAS summary statistics for intelligence by Sniekers et al. Details are summarized in **Supplementary Table 1** (21, 22). GWAS summary statistics for male, female, and overall BMI were obtained from the Genetic Investigation of ANthropometric Traits (GIANT) consortium; T1D data were obtained from a large meta-analysis by Chiou et al.; and T2D data were obtained from a meta-analysis of DIAbetes Genetics Replication And Meta-analysis (DIAGRAM), Genetic Epidemiology Research on Adult Health and Aging (GERA) and UK Biobank (23–26). We restricted analyses to autosomal variants with minor allele frequency ≥0.01. All GWAS datasets were based on participants of European ancestry.

### RNA-seq data

The RNA-seq data used for cell type specific analyses were from the study from Finucane et al., which contained gene expression data of 53 human tissues from Genotype-Tissue Expression (GTEx), 152 human tissues published by the Franke lab, and three brain cell types from Cahoy et al. including purified neurons, astrocytes and oligodendrocytes from mouse forebrain (27–31).

### Brain QTL data

Brain expression quantitative trait loci (eQTL) data came from a meta-analysis of six studies by Qi et al., including GTEx, the CommonMind Consortium (CMC), Religious Orders Study and Memory and Aging Project (ROSMAP), the Brain eQTL Almanac project (Braineac), the Architecture of Gene Expression (CAGE), and eQTLGen (28, 32–36). The brain meta-eQTL data contained 1194 samples, and only SNPs within 1Mb distance from each probe were used.

### Genetic correlation analysis

We implemented cross-trait linkage disequilibrium score (LDSC) regression to evaluate genetic correlation of each education-related phenotype (educational attainment, cognitive performance and intelligence) with each BMI phenotype (male BMI, female BMI and overall BMI) or each diabetes (T1D and T2D) (37). We investigated the SNP heritability and sample overlap while performing genetic correlation analysis. The statistically significant association after adjusting for multiple testing is defined as *P* < 0.05/15 = 0.0033.

### Univariable MR

MR analyses used independent genome-wide significant SNPs (P < 5E-08, r^2^ < 0.3) as exposure instruments. Causal estimates were obtained using the inverse variance weighted (IVW) method. MR-Egger, weighted median, simple mode, and weighted mode analyses were used as sensitivity analyses. The HEIDI-outlier procedure in generalized summary-data-based MR (GSMR) was used to reduce pleiotropic outliers. Directionality and robustness were assessed using instrument-strength statistics, leave-one-out IVW, Steiger directionality filtering, residual global and outlier diagnostics, and stricter exposure P-value threshold analyses (P < 1E-08 and P < 5E-09). Reverse MR analyses evaluated BMI and diabetes as exposures and education-related phenotypes as outcomes.

The TwoSampleMR R package was used for IVW and MR-Egger analyses. The GCTA software package was used for GSMR analysis. Multiple-testing significance for univariable MR was defined as P < 0.05/15 = 0.0033.

### Multivariable MR

Given the genetic overlap across educational attainment, cognitive performance, and intelligence, multivariable MR was used to estimate independent associations of these education-related phenotypes (15, 16, 21, 38, 39). When the three phenotypes were simultaneously modeled as exposures, independent SNPs significantly associated with at least one exposure were selected as instruments and linear MR was performed (40, 41). The TwoSampleMR R package was used for multivariable MR. Statistical significance was defined as P < 0.05.

### Tissue and cell-type specific enrichment

Tissue- and cell-type-specific enrichment was evaluated for educational attainment, cognitive performance, intelligence, BMI, and diabetes using stratified linkage disequilibrium score regression (S-LDSC) (27). SNP signals were mapped to regions surrounding genes with the highest specific expression in 208 tissues and cell types. Significance after multiple testing was defined as P < 0.05/208 = 2.40E-04.

### Identification of candidate causal genes

Candidate genes were evaluated using summary-data-based MR (SMR), which integrates GWAS phenotype associations with eQTL associations in specific tissues (42). The HEIDI-outlier test was used to distinguish linkage from causality or pleiotropy (42). Genes passing both SMR testing (Bonferroni-corrected P < 0.05/number of probes) and HEIDI-outlier testing (P > 0.05 with >10 SNPs) were summarized as candidate genes.

Susceptibility SNP signals were mapped to cis-eQTL target genes and linked to GWAS phenotypes. LD was estimated using the 1000 Genomes Project reference panel, and significant eQTL expression probes (P < 5E-08) were analyzed.

### Overlapping susceptibility loci for joint phenotypes

Overlapping susceptibility loci for educational attainment-BMI and educational attainment-T2D joint phenotypes were evaluated using MTAG and CPASSOC (43, 44). Candidate joint-phenotype loci were defined as independent genome-wide significant SNPs in MTAG (LD r < 0.1, P < 5E-08), passing CPASSOC sensitivity testing (P < 5E-08), and not detected in either single trait. Gene-set enrichment analyses for joint phenotypes were conducted in 53 GTEx tissue types using MAGMA and over-representation enrichment analyses in Gene Ontology and Kyoto Encyclopedia of Genes and Genomes (28, 45–47).

### Transcriptome-wide association analysis

S-PrediXcan TWAS was performed with MetaXcan v0.7.5 using GWAS summary statistics and GTEx v8 MASHR expression-prediction models for 13 brain tissues. Benjamini-Hochberg FDR correction was applied within each trait across all tested gene-tissue pairs. Bonferroni-corrected results and multi-SNP prediction-model signals were summarized.

## Results

### Study population in UK Biobank

The baseline and cross-sectional UK Biobank cohorts included 501,932 participants, and the prospective cohort included 474,659 participants after exclusion of baseline diabetes (**Supplementary Table 2**). Mean age was 56.5 years in the cross-sectional cohort and 56.4 years in the prospective cohort; 45.6% and 44.8% were male, respectively. Higher educational attainment was present in 32.1% of the cross-sectional cohort and 32.6% of the prospective cohort. Prevalent T1D and T2D were recorded in 2,683 (0.535%) and 13,718 (2.733%) participants, respectively. In the prospective cohort, 772 participants developed incident T1D (0.163%) and 25,239 developed incident T2D (5.317%).

### Educational attainment and prevalent diabetes in UK Biobank

**Figure 1:**
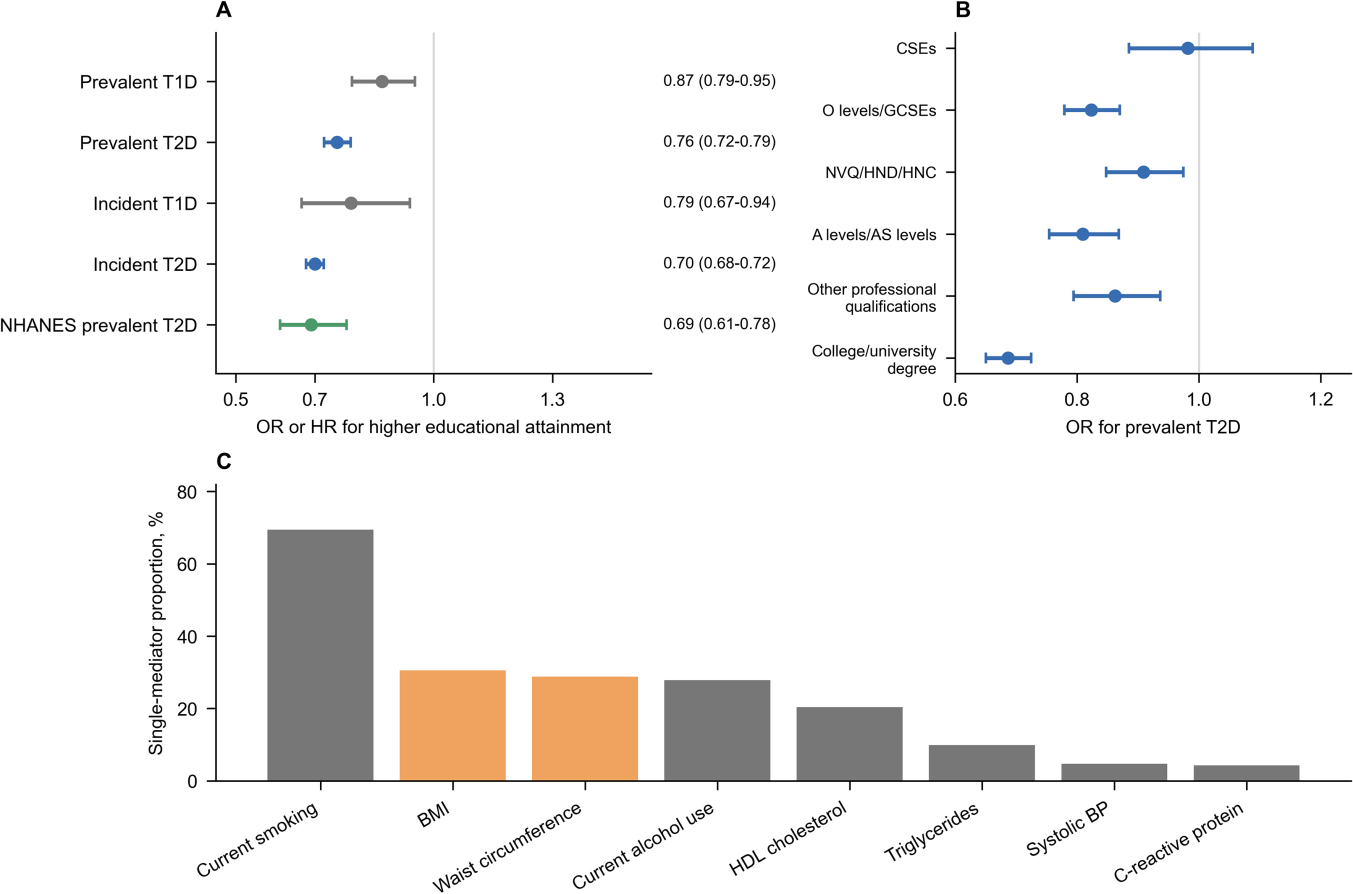
Observational and validation evidence for the association between educational attainment and T2D. **(A)** Fully adjusted UK Biobank associations with prevalent and incident diabetes and NHANES validation for prevalent T2D. Blue markers indicate UK Biobank T2D estimates, gray markers indicate UK Biobank T1D record estimates, and green markers indicate NHANES T2D estimates. **(B)** Detailed UK Biobank qualification categories and prevalent T2D odds relative to no qualifications. **(C)** Single-mediator proportions for incident T2D in UK Biobank. Orange bars indicate adiposity measures.

Higher educational attainment was inversely associated with prevalent T2D in UK Biobank. In Models 1-3, ORs for prevalent T2D were 0.60 (95% CI 0.57-0.62), 0.63 (0.60-0.65), and 0.76 (0.72-0.79), respectively (**Supplementary Table 3**; **Figure 1A**). Detailed education-category analyses showed lower odds of prevalent T2D across most qualification categories relative to no qualifications, including college/university degree (OR 0.69, 95% CI 0.65-0.72; **Figure 1B**; **Supplementary Table 4**). Prevalent T1D estimates were also inverse in the primary models (Model 3 OR 0.87, 95% CI 0.79-0.95), but T1D and T2D first-occurrence records overlapped in 4,358 participants, so T1D analyses are reported as secondary diabetes-record analyses (**Supplementary Figure 1**; **Supplementary Tables 5**–**7**).

### Educational attainment and incident diabetes in UK Biobank

In prospective UK Biobank analyses, higher educational attainment was associated with lower incident T2D risk. Across Models 1-3, HRs for incident T2D were 0.55 (95% CI 0.53-0.57), 0.59 (0.57-0.61), and 0.70 (0.68-0.72), respectively (**Table 1**; **Figure 1A**). Incident T1D estimates were inverse in primary models (Model 3 HR 0.79, 95% CI 0.67-0.94), but after excluding participants with any T2D first-occurrence record, the incident T1D estimate was HR 1.02 (95% CI 0.77-1.35; **Supplementary Table 7**). Early and late follow-up analyses showed similar incident T2D estimates (HR 0.70 in both time windows; **Supplementary Table 8**).

**Table 1:** Associations of higher educational attainment with incident T1D and T2D in the prospective UK Biobank cohort.

Outcome	Model	Beta	SE	HR	CI lower	CI upper	P value
Event T1D	Model 1	-0.4231	0.0842	0.6550	0.5553	0.7726	5.11E-07
Event T1D	Model 2	-0.3333	0.0871	0.7166	0.6042	0.8499	0.000129
Event T1D	Model 3	-0.2340	0.0875	0.7913	0.6666	0.9394	0.007481
Event T2D	Model 1	-0.5961	0.0156	0.5509	0.5344	0.5680	< 1E-100
Event T2D	Model 2	-0.5264	0.0161	0.5907	0.5724	0.6096	1.2E-235
Event T2D	Model 3	-0.3572	0.0161	0.6997	0.6779	0.7221	4.8E-109

Hazard ratios and 95% confidence intervals were estimated using Cox proportional hazards regression. Model 1 was adjusted for age, sex, and ethnicity. Model 2 was additionally adjusted for current smoking, current alcohol use, and physical activity. Model 3 was additionally adjusted for body mass index.

### Potential mediators of the association between educational attainment and T2D

Single-mediator analyses for incident T2D showed attenuation through multiple correlated pathways. Current smoking accounted for the largest single-mediator proportion (69.4%), followed by BMI (30.5%), waist circumference (28.8%), current alcohol use (27.9%), HDL cholesterol (20.4%), triglycerides (9.9%), systolic blood pressure (4.7%), and C-reactive protein (4.3%) (**Table 2**; **Figure 1C**). In the joint mediator-adjusted model, the HR for higher educational attainment and incident T2D was 0.73 (95% CI 0.71-0.75), compared with 0.70 (0.68-0.72) after adjustment for lifestyle factors and BMI alone (**Supplementary Table 9**).

**Table 2:** Mediation of the association between higher educational attainment and T2D by adiposity, lifestyle factors, and metabolic biomarkers.

Mediator	A path	B path	Total effect	Direct effect	Effect	CI lower	CI upper	Proportion mediated	Analysis
BMI	-1.1390	0.1532	-0.5711	-0.3992	-0.1744	-0.1792	-0.1693	0.3055	Prospective
Current smoking	-0.8248	0.4807	-0.5711	-0.5434	-0.3965	-0.4391	-0.3564	0.6943	Prospective
Current alcohol use	0.3170	-0.5019	-0.5711	-0.5605	-0.1591	-0.1793	-0.1397	0.2786	Prospective
Waist circumference	-2.4417	0.0673	-0.5711	-0.4132	-0.1644	-0.1696	-0.1596	0.2878	Prospective
SBP	-2.2083	0.0122	-0.5711	-0.5464	-0.0269	-0.0291	-0.0248	0.0471	Prospective
Triglycerides	-0.1318	0.4293	-0.5711	-0.5197	-0.0566	-0.0592	-0.0537	0.0991	Prospective
HDL cholesterol	0.0542	-2.1510	-0.5711	-0.4886	-0.1166	-0.1222	-0.1113	0.2042	Prospective
CRP	-0.5184	0.0470	-0.5711	-0.5470	-0.0244	-0.0258	-0.0228	0.0427	Prospective
BMI	-1.2023	0.1476	-0.4915	-0.3051	-0.1775	-0.1829	-0.1722	0.3611	Cross-sectional
Current smoking	-0.8168	0.0741	-0.4915	-0.4879	-0.0605	-0.1182	-0.0068	0.1231	Cross-sectional
Current alcohol use	0.3370	-0.7899	-0.4915	-0.4703	-0.2662	-0.2908	-0.2406	0.5416	Cross-sectional
Waist circumference	-2.6194	0.0665	-0.4915	-0.3115	-0.1743	-0.1796	-0.1690	0.3546	Cross-sectional

Indirect effects were approximated as the product of the exposure-to-mediator effect and the mediator-to-outcome effect on the model link scale. Confidence intervals for indirect effects were obtained by bootstrap resampling.

Cross-sectional mediation analyses for prevalent T2D showed similar attenuation patterns: BMI accounted for 36.1% of the association, waist circumference for 35.5%, and current alcohol use for 54.2%. The joint mediator-adjusted model for prevalent T2D yielded an OR of 0.79 (95% CI 0.75-0.82), compared with 0.77 (0.73-0.80) after adjustment for lifestyle factors and BMI alone (**Supplementary Table 9**).

### Stratified analyses and prediction performance in UK Biobank

In age-stratified analyses, higher educational attainment was associated with lower incident T2D risk in both age groups. In fully adjusted models, HRs were 0.68 (95% CI 0.65-0.71) among participants aged <60 years and 0.73 (0.69-0.76) among those aged >=60 years (**Table 3**). In BMI-stratified analyses, HRs were 0.68 (0.65-0.72) for BMI <30 kg/m2 and 0.77 (0.74-0.81) for BMI >=30 kg/m2. A prediction model incorporating educational attainment and standard clinical/lifestyle features yielded an AUC of 0.808 and Brier score of 0.182 (**Supplementary Table 10**).

**Table 3:** Stratified associations of higher educational attainment with incident T2D by age group and BMI group.

Subgroup	Level	Model	N	Events	HR	CI lower	CI upper	P value	Beta	SE
Age	>=60	Model 1	201357	13539	0.5930	0.5676	0.6195	1.7E-121	-0.5226	0.0223
Age	>=60	Model 2	188289	12213	0.6249	0.5975	0.6537	2.84E-93	-0.4701	0.0229
Age	>=60	Model 3	188289	12213	0.7270	0.6950	0.7606	1.32E-43	-0.3188	0.0230
Age	<60	Model 1	273302	11700	0.5141	0.4927	0.5364	1.2E-206	-0.6654	0.0217
Age	<60	Model 2	262298	10630	0.5593	0.5352	0.5844	7.3E-148	-0.5811	0.0224
Age	<60	Model 3	262298	10630	0.6754	0.6463	0.7059	4.78E-68	-0.3924	0.0225
BMI	<30	Model 1	364754	11613	0.5700	0.5457	0.5954	8.2E-141	-0.5621	0.0222
BMI	<30	Model 2	348461	10566	0.6199	0.5926	0.6484	2.69E-96	-0.4782	0.0230
BMI	<30	Model 3	348461	10566	0.6841	0.6539	0.7156	3.77E-61	-0.3797	0.0230
BMI	>=30	Model 1	109905	13626	0.7152	0.6852	0.7465	3.63E-53	-0.3352	0.0218
BMI	>=30	Model 2	102126	12277	0.7510	0.7186	0.7849	4.04E-37	-0.2863	0.0225
BMI	>=30	Model 3	102126	12277	0.7736	0.7402	0.8084	3.44E-30	-0.2568	0.0225

Hazard ratios and 95% confidence intervals were estimated using Cox proportional hazards regression in strata defined by age (<60 years vs ≥60 years) and body mass index (<30 kg/m² vs ≥30 kg/m²).

### External NHANES validation

The NHANES validation cohort included 21,750 adults from 2011-2018 (**Supplementary Figure 2**). Weighted mean age was 47.6 years, 48.1% were male, weighted mean BMI was 29.2 kg/m2, and weighted prevalent T2D was 9.8% (**Table 4**). Compared with adults without a college degree, college graduates had lower weighted BMI (27.9 vs. 29.8 kg/m2), lower current smoking prevalence (7.6% vs. 24.1%), and lower prevalent T2D prevalence (6.7% vs. 11.2%). In the adjusted weighted logistic model, college graduation or above was associated with lower odds of prevalent T2D (OR 0.69, 95% CI 0.61-0.78; n=20,502; **Supplementary Table 11**; **Figure 1A**).

**Table 4:** Baseline characteristics of the NHANES 2011–2018 validation cohort by educational attainment.

Group	Unweighted n	Weighted age, years	Weighted male, %	Weighted BMI, kg/m2	Weighted current smoking, %	Weighted prevalent T2D, %
Overall	21750	47.6 (17.1)	48.1	29.2 (7.0)	19	9.8
Less than college graduate	16357	47.5 (17.7)	48.1	29.8 (7.2)	24.1	11.2
College graduate or above	5393	47.8 (16.0)	48	27.9 (6.2)	7.6	6.7

### Genetic correlation between education-related traits and metabolic phenotypes

Cross-trait LDSC regression showed significant genetic correlations of educational attainment, cognitive performance, and intelligence with BMI-related traits and diabetes phenotypes (**Supplementary Figure 3**; **Supplementary Tables 12**, **13**). Among the education-related phenotypes, educational attainment showed the strongest inverse genetic correlation with BMI (rg = -0.268, P = 9.27E-91), compared with cognitive performance (rg = -0.123, P = 5.46E-15) and intelligence (rg = -0.144, P = 1.43E-20). The pattern of genetic correlation with T2D was directionally similar to that observed for BMI, whereas the correlation with T1D was weaker.

### Univariable Mendelian randomization

**Figure 2:**
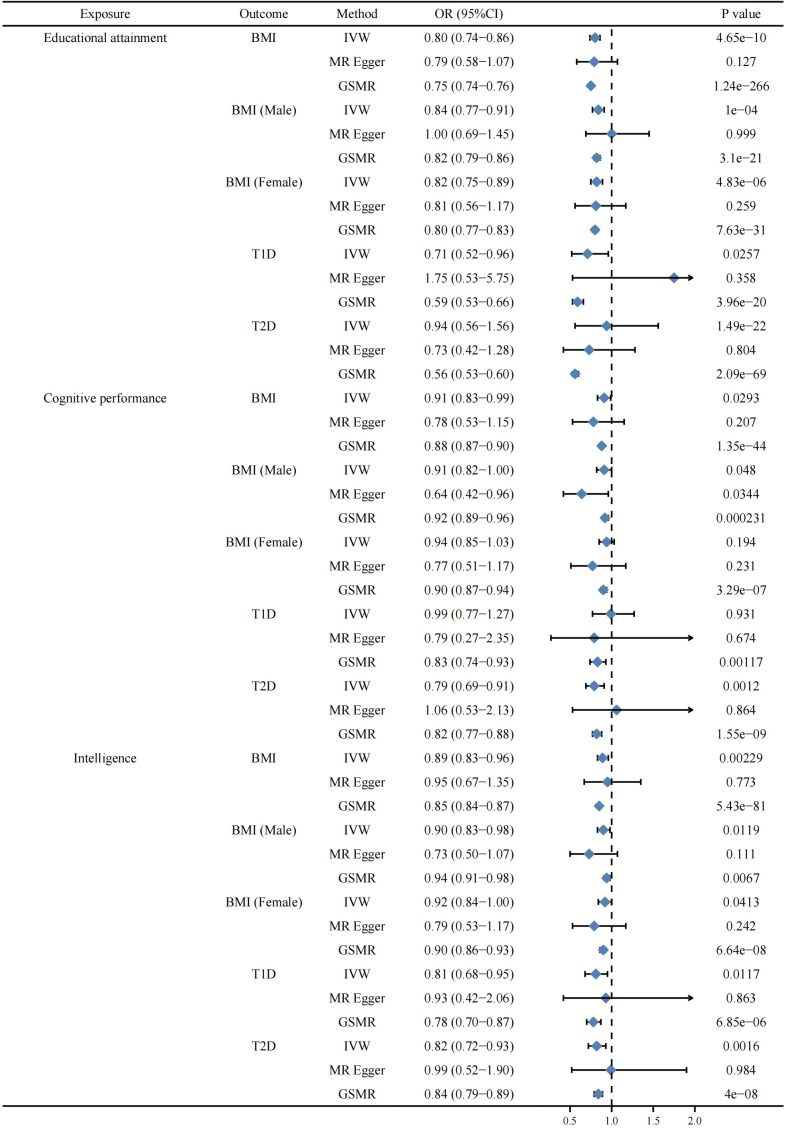
Univariable MR estimates for educational attainment, cognitive performance, and intelligence on BMI and diabetes. BMI, body-mass index; T1D, type 1 diabetes; T2D, type 2 diabetes. Multiple-testing significance was defined as P < 0.05/15 = 0.0033.

Genetically predicted educational attainment, cognitive performance, and intelligence were inversely associated with BMI and T2D in IVW analyses (**Figure 2**; **Supplementary Table 14**). Among education-related phenotypes, educational attainment showed the strongest association with BMI: each 3.61-year increase in educational attainment was associated with lower BMI-related risk (IVW OR 0.80, 95% CI 0.74-0.86; P = 4.65E-10). Instrument diagnostics showed strong instruments for key analyses (minimum F statistic >29), and leave-one-out IVW analyses did not reverse the direction of educational-attainment estimates for BMI, T1D, or T2D (**Supplementary Table 15**). Steiger filtering retained 140 of 141 educational-attainment-to-BMI instruments and 176 of 248 educational-attainment-to-T2D instruments; the Steiger-filtered T2D estimate remained inverse (OR 0.75, 95% CI 0.68-0.82; P = 2.31E-09). Residual outlier diagnostics and stricter exposure P-value thresholds also retained inverse estimates for educational attainment on BMI and T2D (**Supplementary Tables 16**–**18**; **Supplementary Figure 4**).

### Multivariable Mendelian randomization

**Figure 3:**
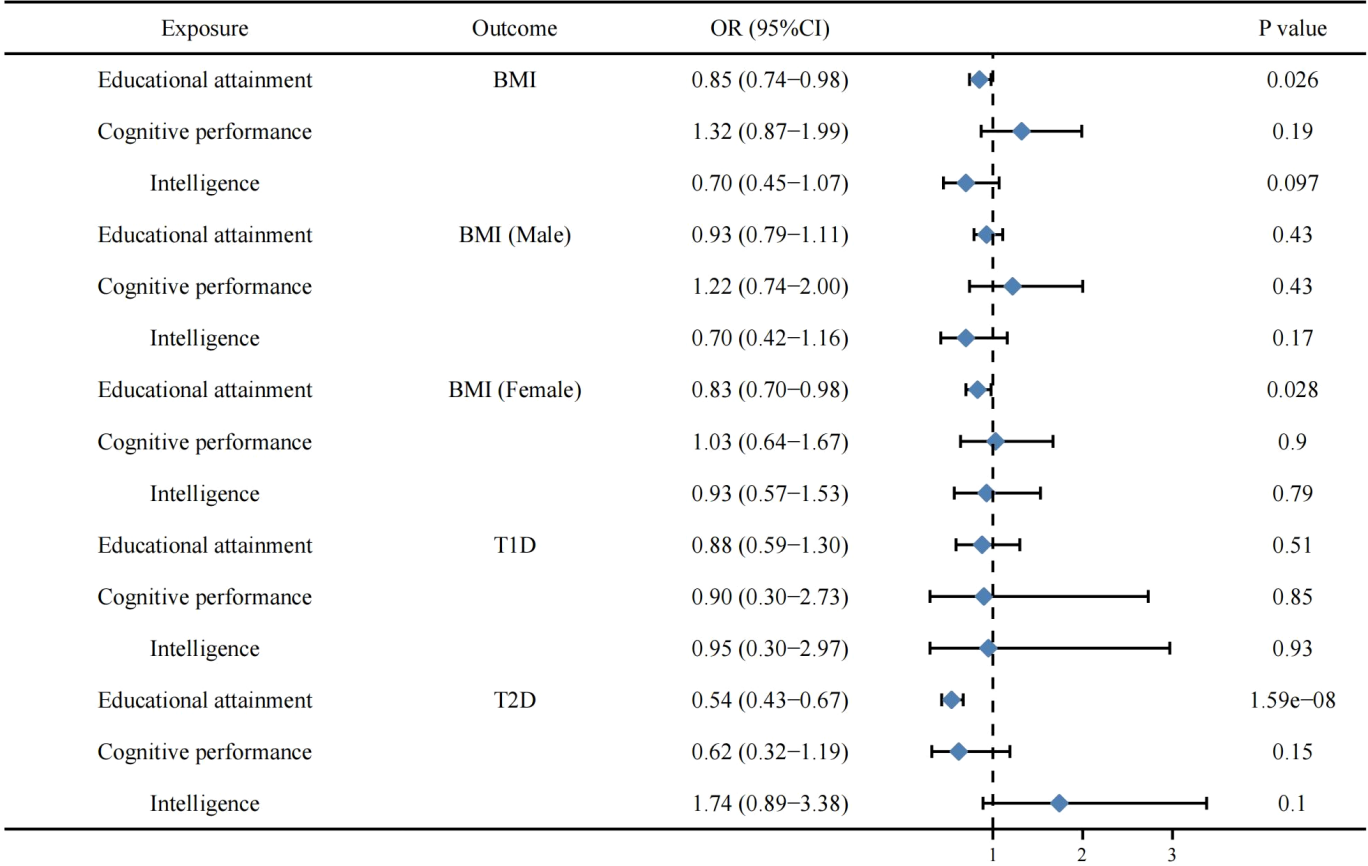
Multivariable MR estimates for educational attainment, cognitive performance, and intelligence on BMI and diabetes. BMI, body-mass index; T1D, type 1 diabetes; T2D, type 2 diabetes.

In multivariable MR analyses, genetically predicted educational attainment remained independently associated with lower BMI (OR 0.83, 95% CI 0.70-0.98; P = 0.028) and lower T2D risk (OR 0.54, 95% CI 0.43-0.67; P = 1.59E-08), whereas cognitive performance and intelligence did not show independent associations (**Figure 3**; **Supplementary Table 19**).

### Reverse causal association

Although genetically predicted BMI was associated with education-related phenotypes, the causal estimates were generally weaker than that of education-related phenotypes on BMI (**Supplementary Figure 5**; **Supplementary Table 20**).

### Tissue- and cell-type-specific enrichment

S-LDSC analyses showed that BMI heritability was enriched in brain tissues, with high enrichment in limbic system, cerebral cortex, and hippocampus (**Supplementary Table 21**). Education-related phenotypes and BMI showed overlapping enrichment in cerebral cortex, limbic system, entorhinal cortex, and hippocampus (**Supplementary Tables 22**–**24**). Neuronal enrichment was observed for BMI and the three education-related phenotypes (**Supplementary Table 25**). T2D showed enrichment patterns closer to BMI and education-related phenotypes than T1D, whereas T1D signals were concentrated in immune tissues and cells (**Supplementary Tables 26**, **27**).

### Shared loci, candidate genes, and pathways

**Figure 4:**
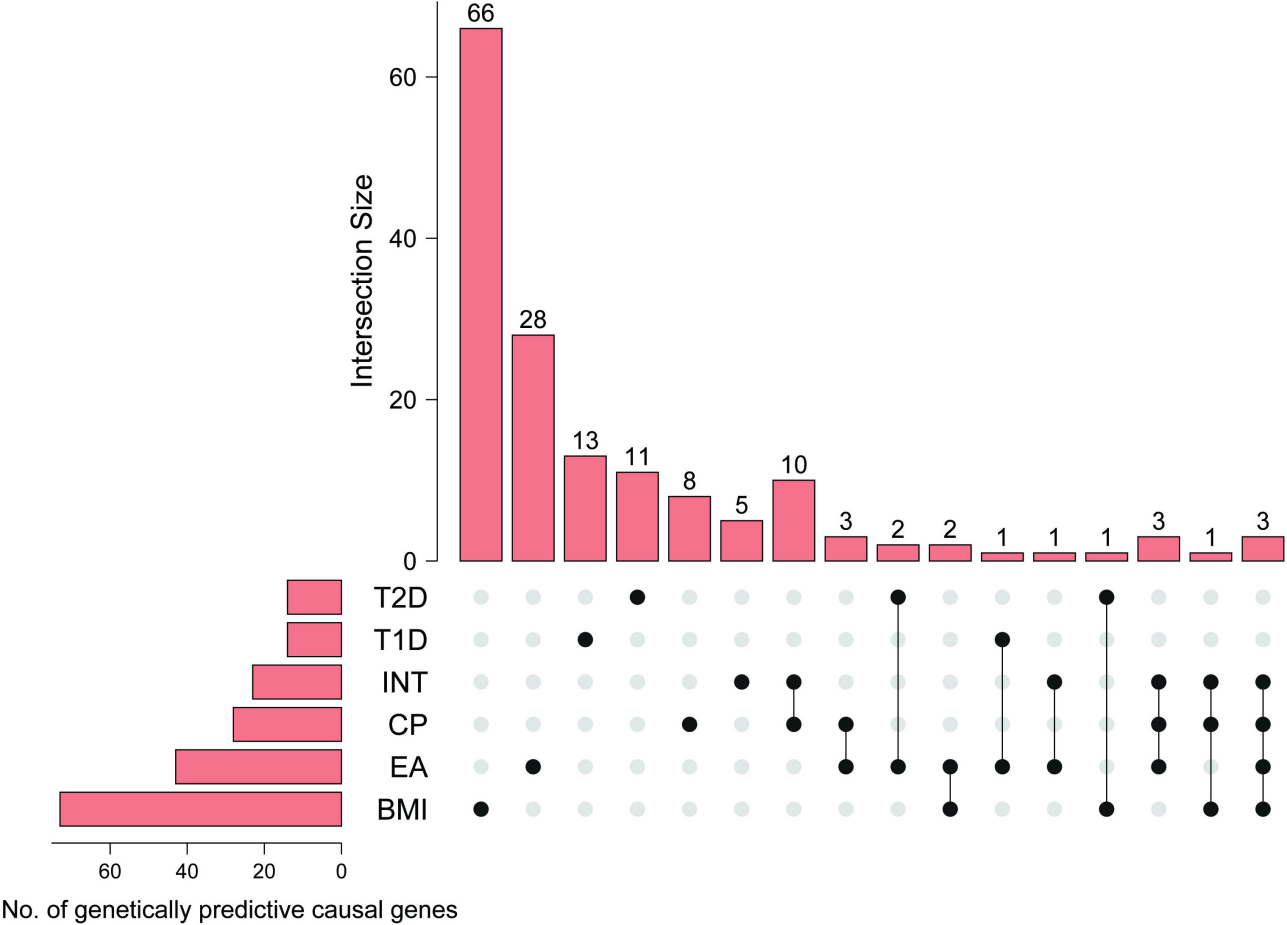
Candidate genes prioritized by SMR for educational attainment, cognitive performance, intelligence, obesity, and diabetes. The left panel shows the number of genes identified by SMR. The right panel shows genes shared by two or more phenotypes.

SMR and HEIDI-outlier analyses identified 73, 43, 28, 23, 14, and 14 candidate genes for educational attainment, cognitive performance, intelligence, BMI, T1D, and T2D, respectively (**Supplementary Table 28**). SULT1A2, ATP5G1, and SNF8 were shared by education-related phenotypes and diabetes-related phenotypes (**Figure 4**).

MTAG and CPASSOC analyses identified 547 independent genome-wide significant SNPs across 399 loci for the educational attainment-BMI joint phenotype and 241 independent genome-wide significant SNPs across 200 loci for the educational attainment-T2D joint phenotype (LD r < 0.1, P < 5E-08; **Supplementary Figures 6**, **7**). Ten candidate joint-phenotype SNPs were not detected in the corresponding single-trait scans (**Table 5**). eQTL annotation linked rs7531118 with NEGR1 expression in brain putamen, brain nucleus accumbens, and brain caudate, and linked rs13188193 with PAM expression in whole blood (**Supplementary Table 29**).

**Table 5:** Candidate SNPs for educational-attainment-BMI and educational-attainment-T2D joint phenotypes using MTAG and CPASSOC.

Trait	SNP	CHR	POS	A1	A2	βEA	PEA	βBMI/T2D	PBMI/T2D	PMTAG	PCPASSOC	Gene
EA-BMI	rs12760039	1	96281780	T	C	0.0139	3.16E-10	0.0156	3.80E-12	6.50E-22	2.79E-20	(intergenic)
	rs7531118	1	72837239	T	C	0.0132	1.99E-14	-0.0256	3.60E-54	7.50E-63	1.28E-57	(intergenic)
	rs12145677	1	110023610	A	G	0.0125	1.32E-11	0.011	1.80E-09	6.92E-21	1.28E-19	SYPL2
	rs9811982	3	49624377	A	C	0.0283	1.44E-53	0.0167	1.10E-16	1.32E-14	1.12E-67	BSN
	rs10939902	5	60727990	T	C	0.0168	4.22E-23	0.0113	4.60E-11	3.38E-34	9.30E-33	ZSWIM6
	rs3814424	5	87968953	T	C	-0.02	1.44E-17	0.03	8.60E-35	7.18E-50	1.46E-45	MEF2C
	rs12189679	6	98333409	A	G	0.0219	7.67E-38	-0.0109	2.60E-10	2.11E-09	1.12E-45	(intergenic)
	rs10241183	7	86230375	T	C	0.0103	5.44E-09	-0.01	3.90E-08	1.56E-16	1.64E-15	GRM3
	rs7868984	9	23357826	T	C	0.0244	1.88E-44	-0.0122	8.50E-10	6.22E-53	1.28E-50	(intergenic)
EA-T2D	rs13188193	5	102754950	T	C	0.0227	5.92E-09	0.1177	2.39E-13	2.36E-20	2.66E-13	(intergenic)

Gene-set analyses of educational attainment-BMI and educational attainment-T2D joint phenotypes showed broad enrichment across GTEx brain tissues (**Supplementary Figure 8**). GO and KEGG analyses prioritized terms related to synaptic organization, neuronal processes, and Hippo signaling (**Supplementary Figure 9**).

### S-PrediXcan transcriptome-wide association analysis

**Figure 5:**
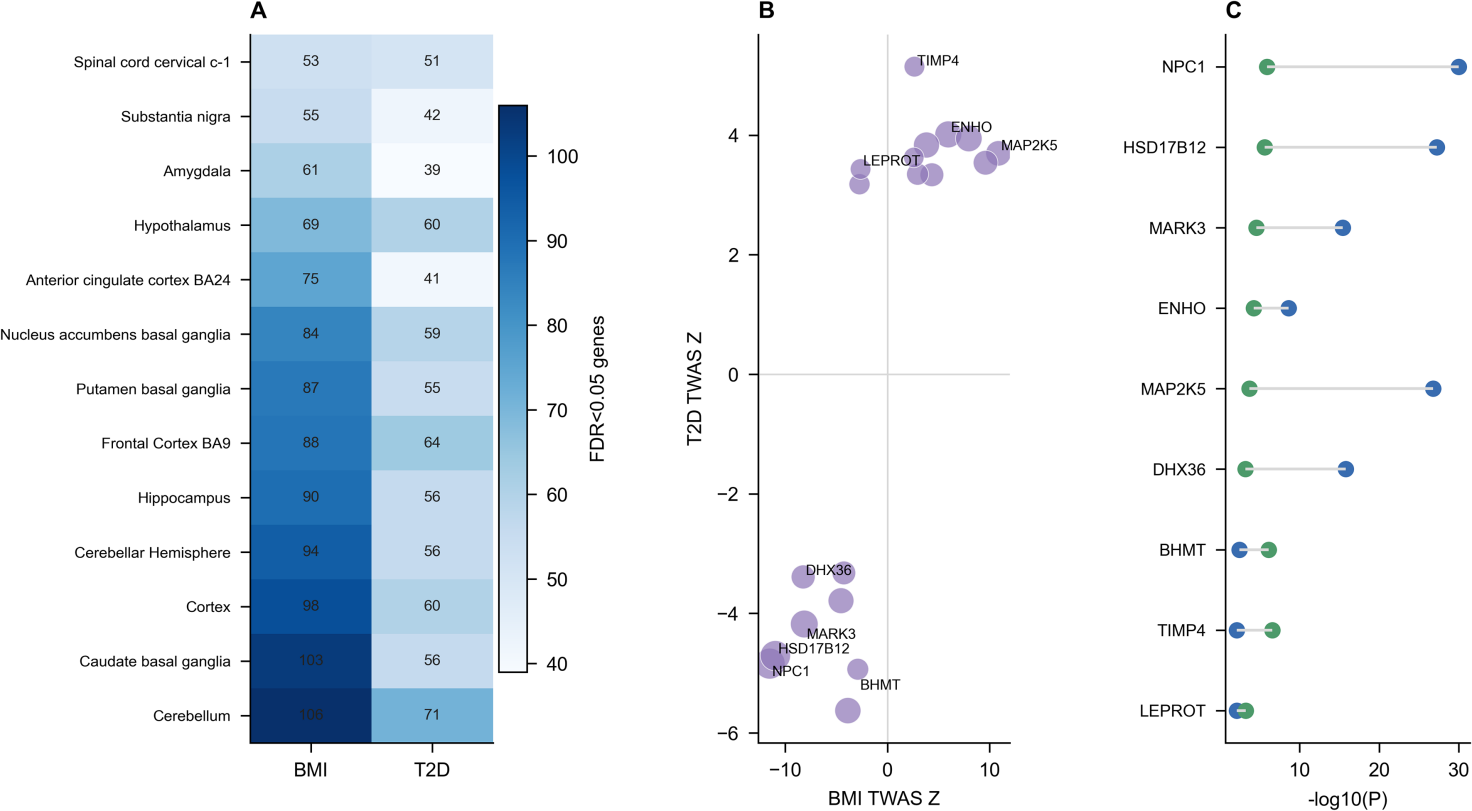
Brain-tissue S-PrediXcan TWAS for BMI and T2D. **(A)** Number of FDR-significant multi-SNP gene signals by GTEx v8 brain tissue model. **(B)** BMI and T2D TWAS Z scores for shared FDR-significant multi-SNP gene symbols. **(C)** Prioritized shared multi-SNP genes ranked by BMI and T2D TWAS P values. Blue markers indicate BMI and green markers indicate T2D.

S-PrediXcan TWAS across 13 GTEx v8 brain-tissue models included 53,265 BMI and 97,192 T2D gene-tissue tests (**Supplementary Tables 30**–**34**). For BMI, 11,254 gene-tissue associations reached FDR<0.05 and 2,419 remained Bonferroni significant. For T2D, 3,600 gene-tissue associations reached FDR<0.05 and 368 remained Bonferroni significant. Across tissues, 332 gene symbols were FDR-significant for both BMI and T2D, including 19 shared signals supported by multi-SNP prediction models. Prioritized shared multi-SNP genes included NPC1, HSD17B12, MARK3, ENHO, MAP2K5, DHX36, BHMT, TIMP4, and LEPROT (**Figure 5**; **Supplementary Table 33**).

## Discussion

This study integrated large-scale observational and genetic evidence to evaluate the relationship between educational attainment and diabetes. The most consistent finding was the inverse association between higher educational attainment and T2D, observed in UK Biobank cross-sectional analyses, UK Biobank prospective analyses, NHANES cross-sectional validation, and complementary genetic analyses.

Second, the mediator analyses showed attenuation of the education-T2D association through adiposity, smoking, alcohol use, lipids, blood pressure, and inflammation. Because these pathways are correlated, the single-mediator proportions are best read as pathway-specific summaries rather than independent additive contributions. The joint mediator-adjusted models showed that the association was attenuated but not eliminated.

Third, the genetic analyses were consistent with an inverse relationship between educational attainment, BMI, and T2D. Educational attainment, cognitive performance, and intelligence were associated with BMI and T2D in univariable MR, but only educational attainment remained independently associated in multivariable MR. This pattern indicates that educational attainment is the education-related phenotype most closely aligned with the observed metabolic associations in these data.

Fourth, tissue enrichment, SMR, shared-locus, and TWAS analyses provided biological context for the epidemiological findings. Brain and neuronal enrichment were prominent for BMI and education-related traits, and the BMI/T2D TWAS identified shared multi-SNP gene signals across brain tissue models (49–57). These findings prioritize candidate tissues and genes for follow-up studies.

These findings have practical implications for diabetes prevention and health policy. Educational attainment itself is shaped by long-term social and structural conditions and is not a clinical intervention in adulthood; however, the consistent association with T2D across UK Biobank, NHANES, and genetic analyses indicates that education-related disadvantage can help identify populations who may benefit from earlier metabolic risk assessment and prevention. The mediator patterns suggest that interventions addressing adiposity, smoking, alcohol use, dyslipidemia, blood pressure, and systemic inflammation may reduce part of the excess T2D burden associated with lower educational attainment. At the policy level, the results support integrated strategies that combine educational opportunity, health literacy, and accessible cardiometabolic prevention rather than focusing only on individual behavior. Future studies should evaluate whether education-sensitive screening pathways, community-based prevention programs, and longitudinal multi-omics studies can clarify when and how social exposures become embedded in metabolic risk.

## Limitations

Several limitations should be considered. First, mediation analyses were primarily single-mediator analyses, and candidate mediators are correlated; joint mediator-adjusted models partly addressed this issue but did not provide formal causal decomposition across multiple mediators. Second, residual confounding remains possible in observational analyses. Third, educational attainment was primarily analyzed as a binary exposure, although detailed qualification categories supported the main T2D finding. Fourth, UK Biobank adult-onset T1D records overlap substantially with T2D records and can include latent autoimmune diabetes in adults, insulin-treated T2D, or diabetes-type misclassification; T1D analyses were therefore secondary. Fifth, UK Biobank is not fully representative of the general UK population, and selection and participation biases can affect absolute estimates (48). Sixth, NHANES provided cross-sectional validation of prevalent T2D only. Seventh, most genetic datasets were based on individuals of European ancestry. Eighth, SMR, shared-locus analyses, and TWAS prioritize candidate genes and tissues but do not prove causality or tissue-specific mechanism without functional validation.

## Conclusions

Higher educational attainment was associated with lower prevalent and incident T2D risk. Adiposity, smoking, alcohol use, and cardiometabolic traits attenuated the education-T2D association. Genetic analyses further aligned educational attainment with lower BMI and T2D risk and prioritized candidate brain-tissue transcriptomic signals shared by BMI and T2D. These results support educational attainment as an upstream social determinant of metabolic health and identify modifiable pathways relevant to T2D prevention.

## Data Availability

Access to the UK Biobank data can be requested through a standard protocol (https://www.ukbiobank.ac.uk/register-apply/). Data used in this study are available in the UK Biobank under application ID 249728. GWAS for educational attainment and cognitive performance: https://thessgac.com/papers/; GWAS for intelligence, T1D, and T2D: https://www.ebi.ac.uk/gwas/; GWAS for BMI: https://portals.broadinstitute.org/collaboration/giant/index.php/GIANT_consortium.
