# Supplementary material for: Educational attainment and diabetes risk: triangulation evidence from UK Biobank prospective cohort, NHANES 2011-2018, and cross-trait genomics analyses
Source: Front Endocrinol (Lausanne). 2026 Jun 26;17:1867159. doi: 10.3389/fendo.2026.1867159 (PMC13350059; doi:10.3389/fendo.2026.1867159)

**Educational attainment and diabetes risk: triangulation evidence from UK Biobank prospective cohort, NHANES 2011-2018, and cross-trait genomics analyses**

Guannan Geng^1^, Shizheng Qiu^2,3^, Zhishuai Zhang^2,3^, Xinru Liu^4^, Xin Wang^1^, Yang Hu^2,3*^, Hongyu Kuang^1*^, Jiahui Zhang^4,5,6*^

^1^Department of Endocrinology, The First Affiliated Hospital of Harbin Medical University, Harbin, China

^2^Center for Bioinformatics, Faculty of Computing, Harbin Institute of Technology, Harbin, China.

^3^Key Laboratory of Biological Bigdata, Ministry of Education, Harbin Institute of Technology, Harbin, China.

^4^Department of Stomatology, The Fourth Hospital of Harbin Medical University, Harbin, China.

^5^Heilongjiang Provincial Key Laboratory of Hard Tissue Development and Regeneration, Harbin, China.

^6^School of Stomatology, Harbin Medical University, Harbin, China.

***Correspondence:**

Yang Hu, Center for Bioinformatics, Faculty of Computing, Harbin Institute of Technology, 92 Xidazhi Street, Nangang District, Harbin, 150001, China. Email: [huyang@hit.edu.cn](mailto:huyang@hit.edu.cn).

Hongyu Kuang, Department of Endocrinology, The First Affiliated Hospital of Harbin Medical University, Harbin, 150001, China. Email: [kuanghongyu@hrbmu.edu.cn](mailto:kuanghongyu@hrbmu.edu.cn)

Jiahui Zhang, Department of Stomatology, The Fourth Hospital of Harbin Medical University, Harbin, China; Heilongjiang Provincial Key Laboratory of Hard Tissue Development and Regeneration, Harbin, China. Email: zjhggn@163.com

Supplementary Figure 1. Diabetes record overlap in UK Biobank. Bars show participants with any T1D first-occurrence date, any T2D first-occurrence date, both T1D and T2D first-occurrence dates, and baseline insulin reporting.


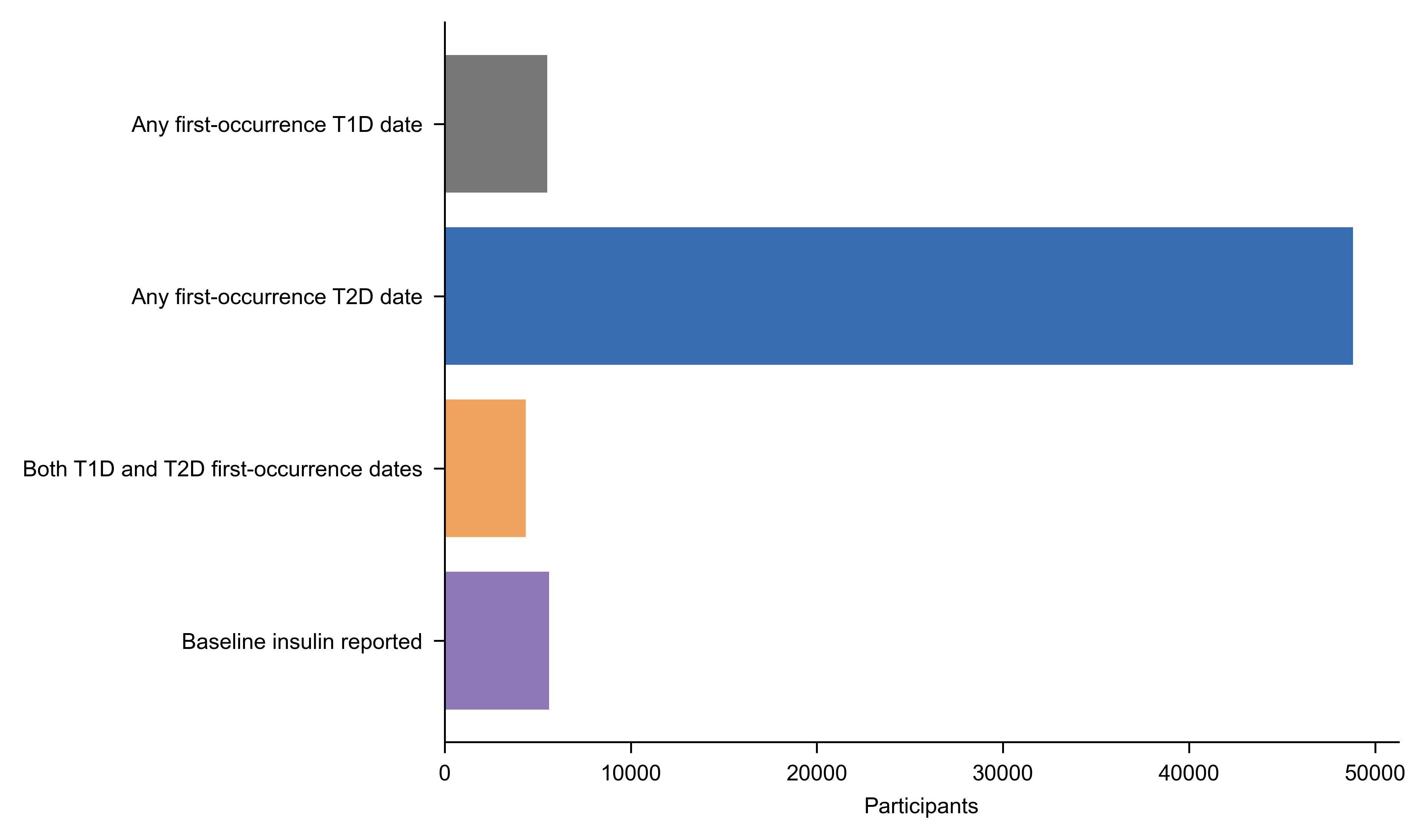


Supplementary Figure 2. NHANES 2011-2018 validation sample by cycle. Grey bars show unweighted participants and the green line shows unweighted prevalent T2D percentage by two-year cycle.


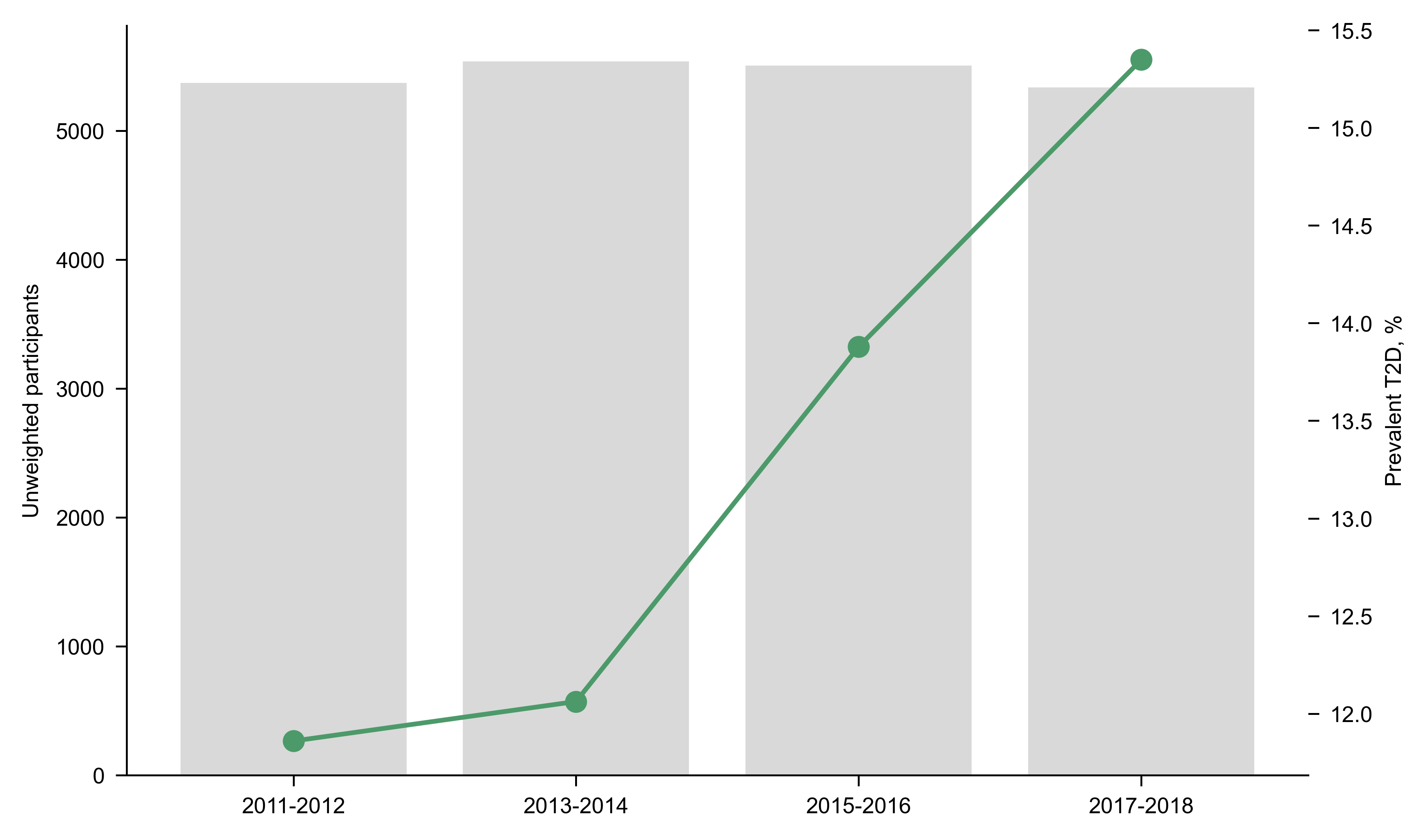


Supplementary Figure 3. Pairwise genetic correlations estimated using LDSC. BMI: body-mass index; CP: cognitive performance; EA: educational attainment; INT: intelligence; genetic correlation: Rg. T1D: type 1 diabetes; T2D: type 2 diabetes.


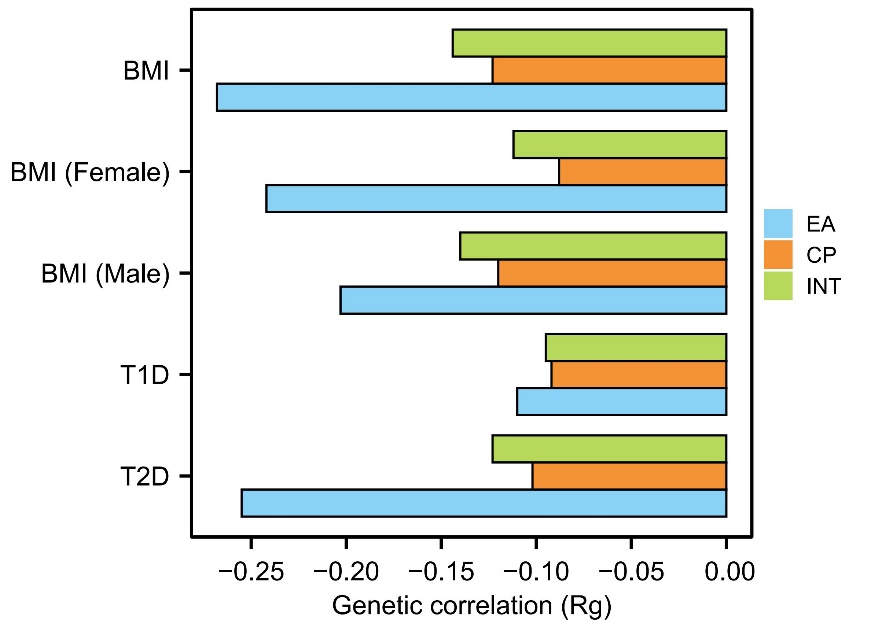


Supplementary Figure 4. MR instrument strength and sensitivity analyses. A, IVW estimates for educational attainment on BMI, T1D, and T2D. B, Minimum instrument F statistics. C, Steiger-filtered and residual outlier-corrected estimates; green markers indicate Steiger-filtered estimates and purple markers indicate outlier-corrected estimates.


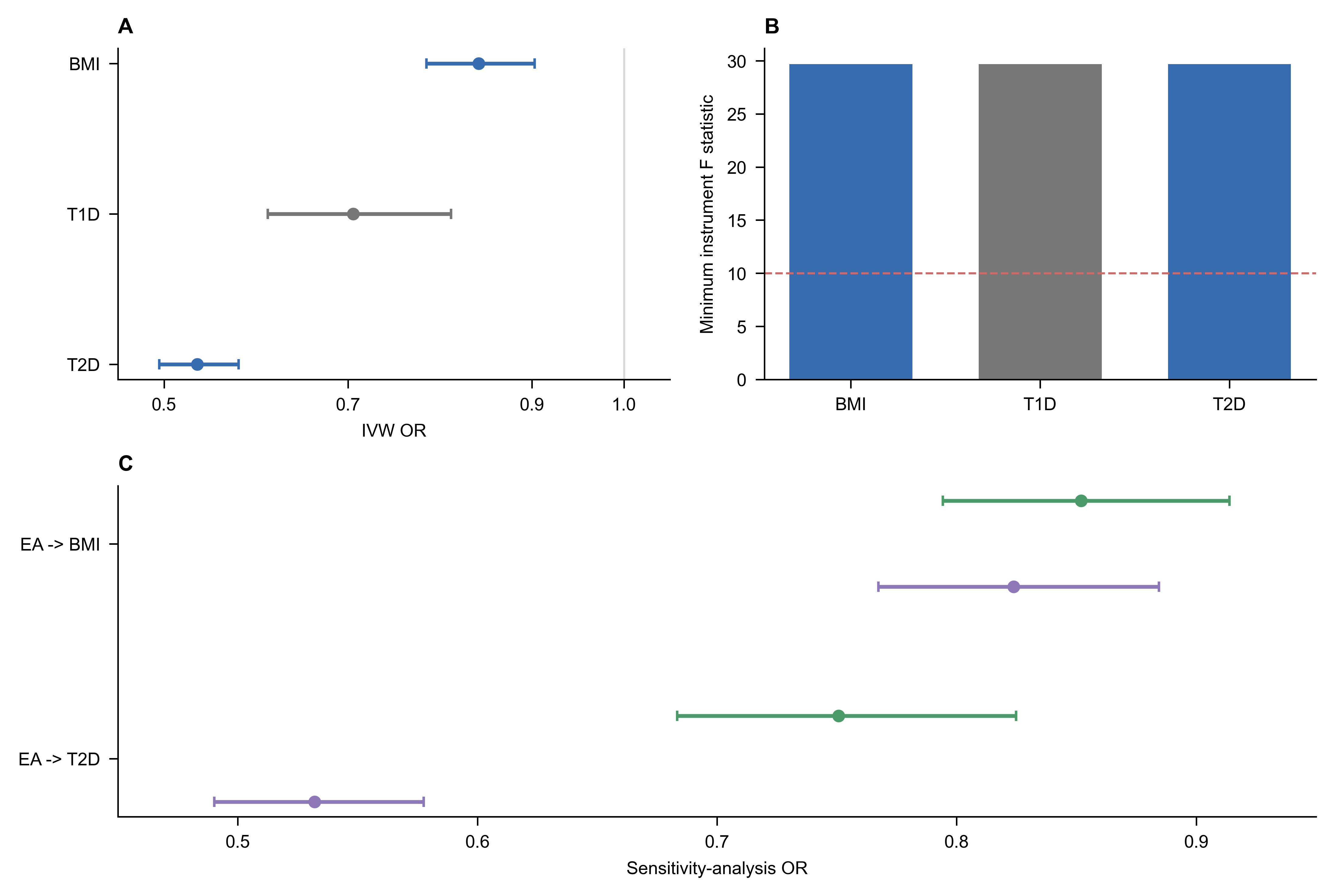


Supplementary Figure 5. Causal estimates of BMI and diabetes on educational attainment, cognitive performance and intelligence using univariable MR analysis.


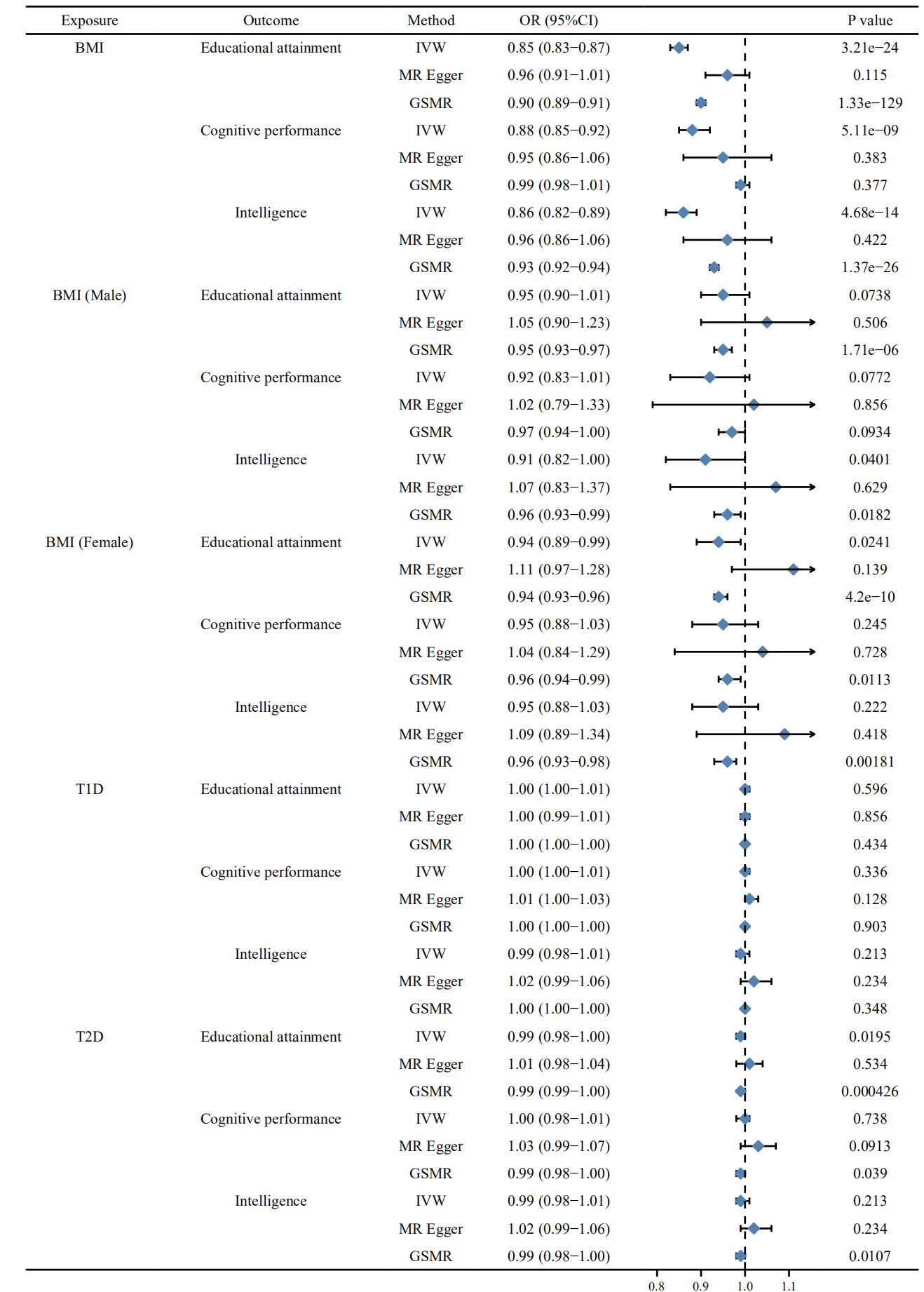


Supplementary Figure 6. Manhattan plots of education-BMI and education-T2D joint phenotypes.


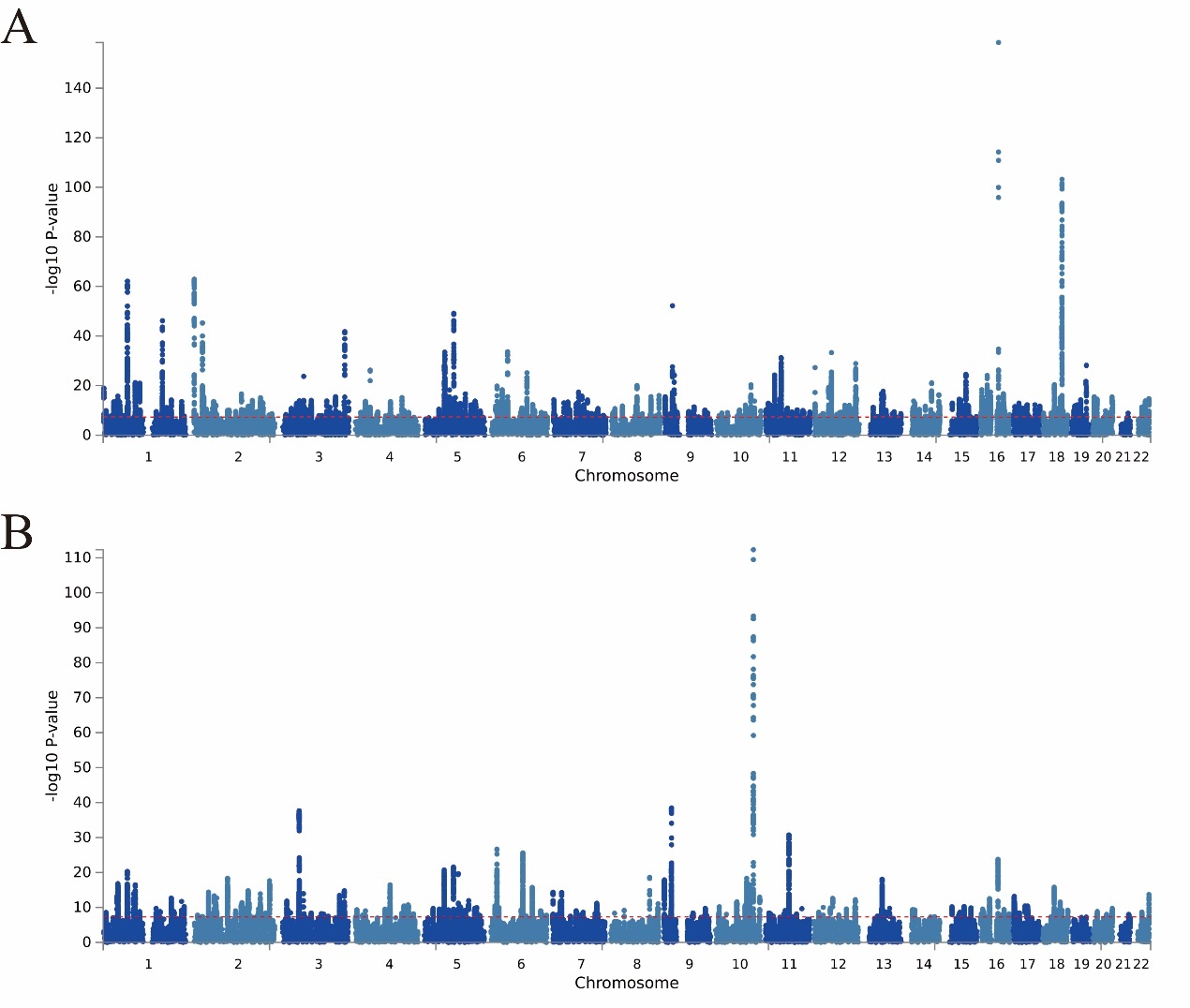


Supplementary Figure 7. Q-Q plots of education-BMI and education-T2D joint phenotypes.


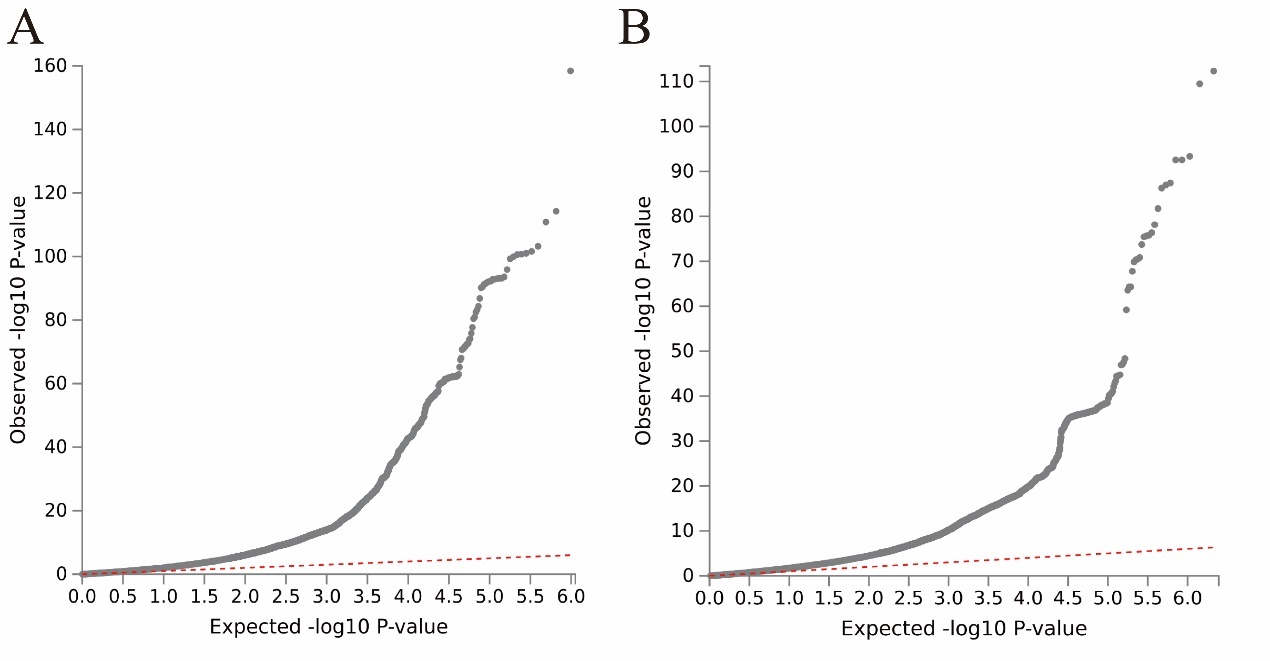


Supplementary Figure 8. Gene set enrichment of education-BMI and education-T2D joint phenotypes in GTEx human tissues.


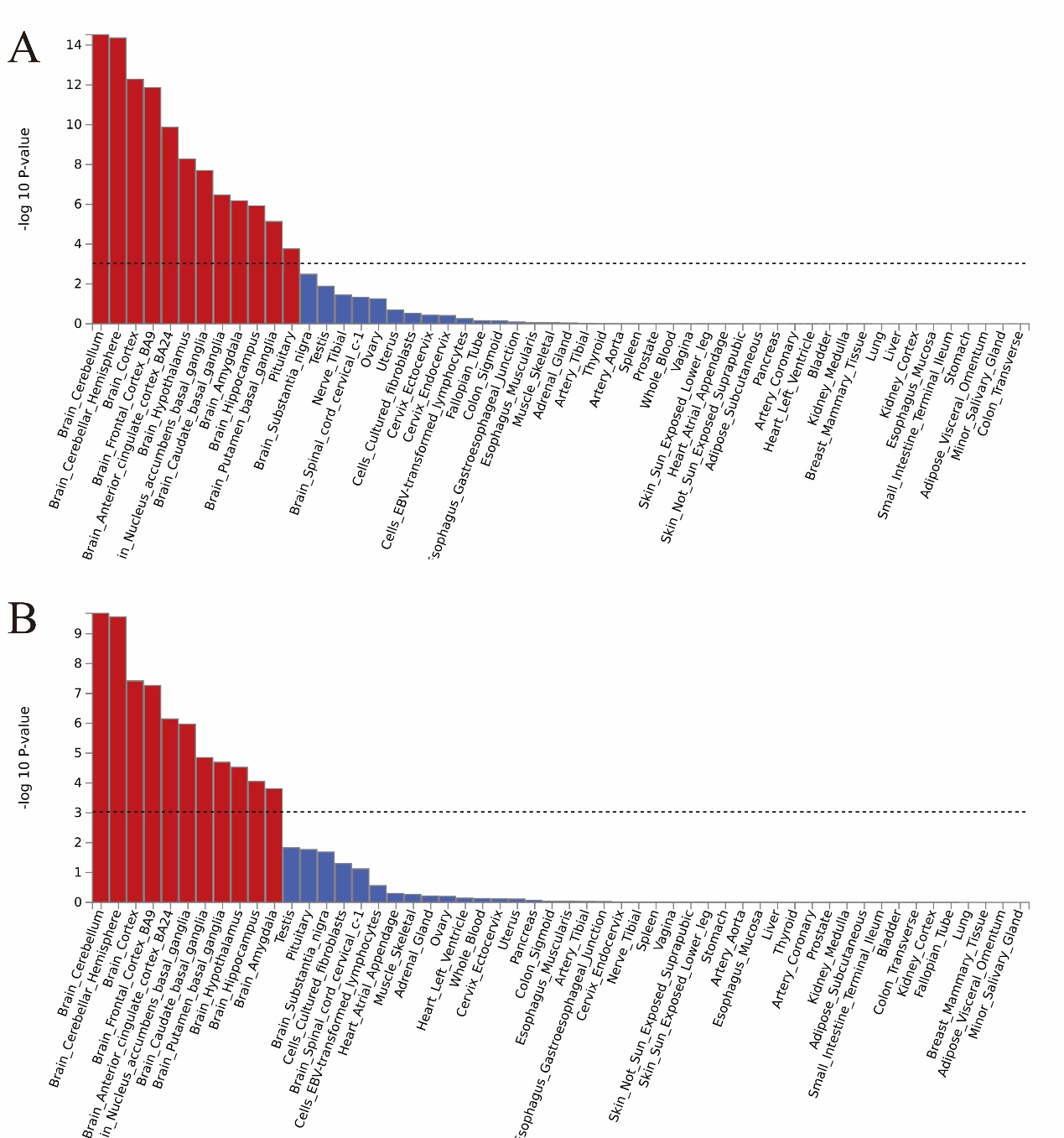


Supplementary Figure 9. GO and KEGG enrichment of education-BMI and education-T2D joint phenotypes.


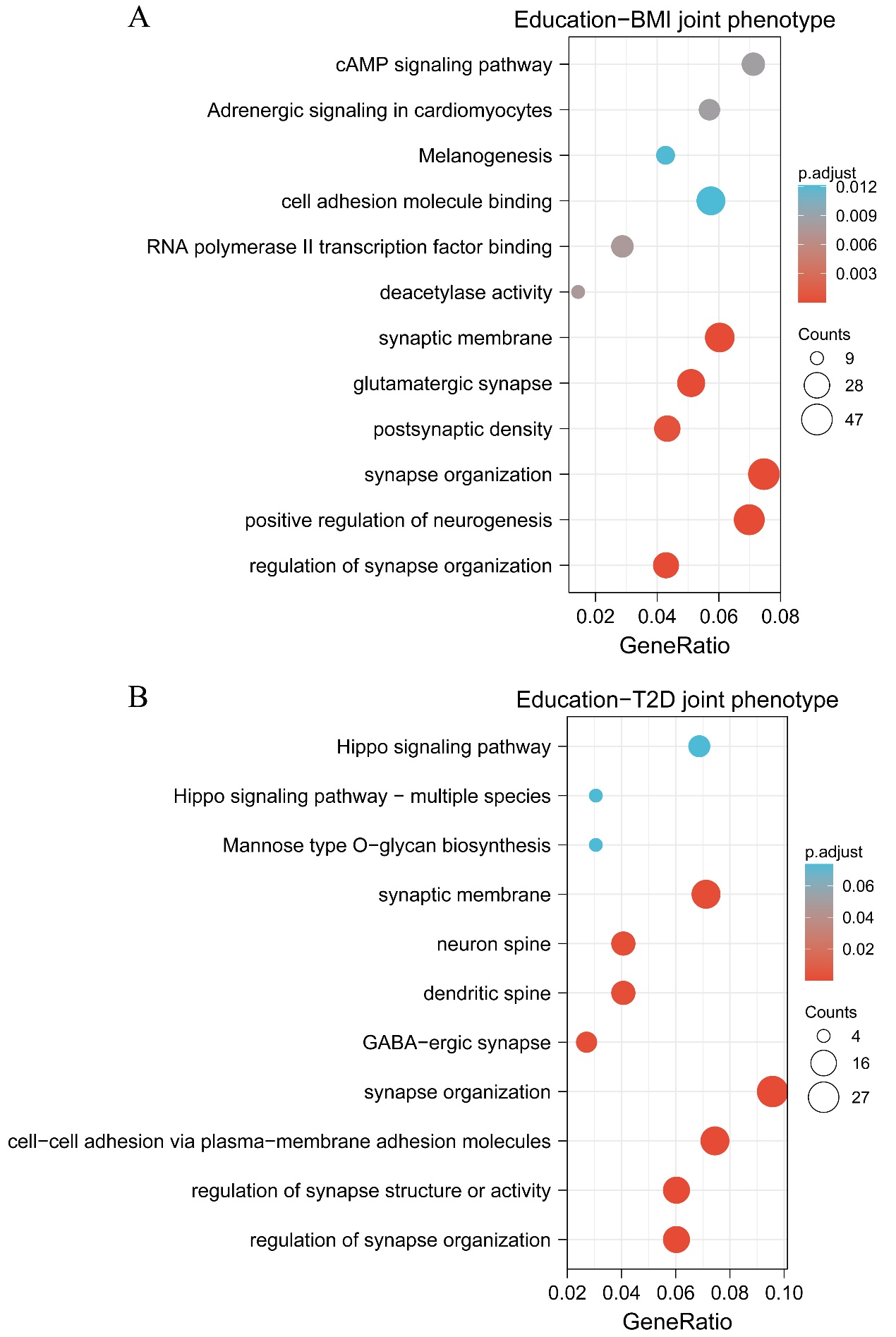

Supplement: Supplementary file 1 [file DataSheet1.docx]
