# Supplementary material for: Educational attainment and diabetes risk: triangulation evidence from UK Biobank prospective cohort, NHANES 2011-2018, and cross-trait genomics analyses
Source: Front Endocrinol (Lausanne). 2026 Jun 26;17:1867159. doi: 10.3389/fendo.2026.1867159 (PMC13350059; doi:10.3389/fendo.2026.1867159)
Supplement: Supplementary file 2 [file DataSheet2.docx]

**Educational attainment and diabetes risk: triangulation evidence from UK Biobank prospective cohort, NHANES 2011-2018, and cross-trait genomics analyses**

Guannan Geng^1^, Shizheng Qiu^2,3^, Zhishuai Zhang^2,3^, Xinru Liu^4^, Xin Wang^1^, Yang Hu^2,3*^, Hongyu Kuang^1*^, Jiahui Zhang^4,5,6*^

^1^Department of Endocrinology, The First Affiliated Hospital of Harbin Medical University, Harbin, China

^2^Center for Bioinformatics, Faculty of Computing, Harbin Institute of Technology, Harbin, China.

^3^Key Laboratory of Biological Bigdata, Ministry of Education, Harbin Institute of Technology, Harbin, China.

^4^Department of Stomatology, The Fourth Hospital of Harbin Medical University, Harbin, China.

^5^Heilongjiang Provincial Key Laboratory of Hard Tissue Development and Regeneration, Harbin, China.

^6^School of Stomatology, Harbin Medical University, Harbin, China.

***Correspondence:**

Yang Hu, Center for Bioinformatics, Faculty of Computing, Harbin Institute of Technology, 92 Xidazhi Street, Nangang District, Harbin, 150001, China. Email: [huyang@hit.edu.cn](mailto:huyang@hit.edu.cn).

Hongyu Kuang, Department of Endocrinology, The First Affiliated Hospital of Harbin Medical University, Harbin, 150001, China. Email: [kuanghongyu@hrbmu.edu.cn](mailto:kuanghongyu@hrbmu.edu.cn)

Jiahui Zhang, Department of Stomatology, The Fourth Hospital of Harbin Medical University, Harbin, China; Heilongjiang Provincial Key Laboratory of Hard Tissue Development and Regeneration, Harbin, China. Email: zjhggn@163.com

Supplementary Table 1. Details of GWAS summary statistics.

| **Trait** | **Source** | **No. of cohorts** | **No. of participants** | **Ethnicity** |
| --- | --- | --- | --- | --- |
| Educational attainment | SSGAC | 71 | 1,131,881 | European |
| Cognitive performance | SSGAC | 71 | 257,828 | European |
| Intelligence | Sniekers et al. | 8 | 78,308 | European |
| BMI | GIANT | 81 | 346, 738 | European |
| Male BMI | GIANT | 80 | 234,069 | European |
| Female BMI | GIANT | 80 | 234,069 | European |
| T1D | Chiou et al. | 21 | 18,942 cases and 501,638 controls | European |
| T2D | Xue et al. | 3 | 12,171 cases and 56,862 controls | European |

BMI: body-mass index; GIANT: Genetic Investigation of ANthropometric Traits Consortium; SSGAC: Social Science Genetic Association Consortium; T1D: type 1 diabetes; T2D: type 2 diabetes.

Supplementary Table 2. Baseline characteristics of participants included in the cross-sectional and prospective analyses in UK Biobank.

| **Characteristics** | **Cross-sectional cohort** | **Prospective cohort** |
| --- | --- | --- |
| N | 501,932 | 474,659 |
| Age | 56.53 ± 8.09 | 56.36 ± 8.11 |
| Male | 228,897 (45.60%) | 212,396 (44.75%) |
| Higher education | 160,931 (32.06%) | 154,566 (32.56%) |
| Other ethnicity | 288,65 (5.75%) | 25,259 (5.32%) |
| Current smoking | 39,206 (7.81%) | 36,923 (7.78%) |
| Current drinking | 460,745 (91.79%) | 437,983 (92.27%) |
| BMI | 27.45 ± 4.79 | 27.23 ± 4.62 |
| Waist circumference | 90.32 ± 13.47 | 89.63 ± 13.07 |
| Physical activity | 1.84 ± 1.96 | 1.85 ± 1.96 |
| SBP | 139.73 ± 19.15 | 139.52 ± 19.18 |
| Prevalent T1D | 2,683 (0.535%) | |
| Prevalent T2D | 13,718 (2.733%) | |
| Healthy control | 402,604 (80.21%) | 402,604 (84.82%) |
| Incident T1D |  | 772 (0.163%) |
| Incident T2D |  | 25,239 (5.317%) |

Data are presented as mean ± standard deviation for continuous variables and number (percentage) for categorical variables. Higher educational attainment was defined as having a college/university qualification or above.

Supplementary Table 3. Associations of higher educational attainment with prevalent T1D and T2D in UK Biobank.

| **analysis** | **outcome** | **model** | **events** | **beta** | **SE** | **OR** | **CI lower** | **CI upper** | **P value** |
| --- | --- | --- | --- | --- | --- | --- | --- | --- | --- |
| Cross-sectional association | T1D | Model 1 | 2683 | -0.27874 | 0.044354 | 0.75674 | 0.693731 | 0.825471 | 3.29E-10 |
| Cross-sectional association | T1D | Model 2 | 2429 | -0.23868 | 0.046188 | 0.787666 | 0.719492 | 0.8623 | 2.37E-07 |
| Cross-sectional association | T1D | Model 3 | 2429 | -0.14009 | 0.046458 | 0.869279 | 0.793621 | 0.952149 | 0.002566 |
| Cross-sectional association | T2D | Model 1 | 13718 | -0.51277 | 0.021454 | 0.598835 | 0.574176 | 0.624554 | 3E-126 |
| Cross-sectional association | T2D | Model 2 | 12285 | -0.46732 | 0.022318 | 0.626679 | 0.599857 | 0.654701 | 2.36E-97 |
| Cross-sectional association | T2D | Model 3 | 12285 | -0.28006 | 0.02273 | 0.75574 | 0.72281 | 0.790171 | 7E-35 |

Odds ratios and 95% confidence intervals were estimated using logistic regression. Model 1 was adjusted for age, sex, and ethnicity. Model 2 was additionally adjusted for current smoking, current drinking, and physical activity. Model 3 was additionally adjusted for body mass index.

Supplementary Table 4. Detailed educational attainment category sensitivity analyses for prevalent diabetes in UK Biobank.

| outcome | education_category | reference | events | Effect (95% CI) | P value |
| --- | --- | --- | --- | --- | --- |
| Baseline T1D | CSEs | None of the above | 2410 | 0.84 (0.68-1.04) | 0.112 |
| baseline T1D | O levels/GCSEs | None of the above | 2410 | 0.80 (0.71-0.90) | 3.65E-04 |
| baseline T1D | NVQ/HND/HNC | None of the above | 2410 | 0.76 (0.65-0.90) | 0.00135 |
| baseline T1D | A levels/AS levels | None of the above | 2410 | 0.73 (0.63-0.85) | 7.70E-05 |
| baseline T1D | Other professional qualifications | None of the above | 2410 | 0.76 (0.63-0.93) | 0.00691 |
| baseline T1D | College/university degree | None of the above | 2410 | 0.72 (0.64-0.81) | 4.20E-08 |
| baseline T2D | CSEs | None of the above | 12247 | 0.98 (0.89-1.09) | 0.726 |
| baseline T2D | O levels/GCSEs | None of the above | 12247 | 0.82 (0.78-0.87) | 6.14E-12 |
| baseline T2D | NVQ/HND/HNC | None of the above | 12247 | 0.91 (0.85-0.97) | 0.00718 |
| baseline T2D | A levels/AS levels | None of the above | 12247 | 0.81 (0.75-0.87) | 4.42E-09 |
| baseline T2D | Other professional qualifications | None of the above | 12247 | 0.86 (0.79-0.94) | 4.37E-04 |
| baseline T2D | College/university degree | None of the above | 12247 | 0.69 (0.65-0.72) | 1.19E-42 |

Supplementary Table 5. UK Biobank diabetes definition and validation counts.

| metric | value |
| --- | --- |
| Any first-occurrence T1D date | 5505 |
| Any first-occurrence T2D date | 48813 |
| Both T1D and T2D first-occurrence dates | 4358 |
| Baseline insulin reported | 5609 |
| Baseline diabetes history by self-report | 26381 |
| Prevalent T1D | 2684 |
| Prevalent T2D | 13873 |
| Incident T1D in prospective cohort | 772 |
| Incident T2D in prospective cohort | 25186 |

Supplementary Table 6. Age at first occurrence for UK Biobank diabetes records.

| Case definition | N | Median age at first occurrence | Q1 | Q3 |
| --- | --- | --- | --- | --- |
| Any T1D first-occurrence date | 5505 | 58.78 | 49.46 | 67.02 |
| Any T2D first-occurrence date | 48813 | 63.85 | 56.74 | 70.28 |
| Prevalent T1D | 2684 | 50.79 | 39.62 | 57.43 |
| Prevalent T2D | 13873 | 56.31 | 50.12 | 60.95 |
| Incident T1D | 2821 | 66.57 | 60.05 | 71.84 |
| Incident T2D | 34940 | 67.29 | 60.99 | 72.36 |

Supplementary Table 7. T1D definition sensitivity analyses excluding participants with any T2D first-occurrence record.

| analysis | n | events | Effect (95% CI) | P value |
| --- | --- | --- | --- | --- |
| Prevalent T1D sensitivity excluding participants with any T2D first-occurrence date | 427850 | 616 | 1.27 (1.08-1.50) | 0.00369 |
| Incident T1D sensitivity excluding participants with any T2D first-occurrence date | 424448 | 228 | 1.02 (0.77-1.35) | 0.875 |

Supplementary Table 8. Early and late follow-up Cox sensitivity analyses for the proportional hazards assumption.

| outcome | window | n | events | Effect (95% CI) | P value |
| --- | --- | --- | --- | --- | --- |
| T1D | Full follow-up | 446964 | 695 | 0.79 (0.66-0.94) | 0.00687 |
| T1D | Early follow-up | 446964 | 356 | 0.86 (0.68-1.09) | 0.221 |
| T1D | Late follow-up | 431088 | 339 | 0.71 (0.55-0.92) | 0.0088 |
| T2D | Full follow-up | 446964 | 22515 | 0.70 (0.68-0.72) | 4.43E-108 |
| T2D | Early follow-up | 446964 | 11191 | 0.70 (0.67-0.74) | 6.43E-52 |
| T2D | Late follow-up | 421787 | 11324 | 0.70 (0.66-0.73) | 4.76E-58 |

Supplementary Table 9. Joint mediator-adjusted models for the education-T2D association.

| analysis | n | events | Effect (95% CI) | P value | model |
| --- | --- | --- | --- | --- | --- |
| Incident T2D Demographic model | 469672 | 24726 | 0.55 (0.54-0.57) | <1E-300 | Demographic model |
| Incident T2D Lifestyle model | 446964 | 22515 | 0.59 (0.57-0.61) | 1.08E-233 | Lifestyle model |
| Incident T2D Lifestyle + BMI model | 446964 | 22515 | 0.70 (0.68-0.72) | 4.43E-108 | Lifestyle + BMI model |
| Incident T2D Joint mediator-adjusted model | 446964 | 22515 | 0.73 (0.71-0.75) | 2.22E-85 | Joint mediator-adjusted model |
| Prevalent T2D Demographic model | 496451 | 13569 | 0.61 (0.58-0.64) | 4.29E-119 | Demographic model |
| Prevalent T2D Lifestyle model | 471331 | 12247 | 0.63 (0.61-0.66) | 2.82E-93 | Lifestyle model |
| Prevalent T2D Lifestyle + BMI model | 471331 | 12247 | 0.77 (0.73-0.80) | 4.55E-32 | Lifestyle + BMI model |
| Prevalent T2D Joint mediator-adjusted model | 471331 | 12247 | 0.79 (0.75-0.82) | 2.54E-26 | Joint mediator-adjusted model |

Supplementary Table 10. Performance of the logistic regression model for predicting incident T2D.

| **Model** | **N** | **Events** | **AUC** | **PR AUC** | **Brier score** | **features** |
| --- | --- | --- | --- | --- | --- | --- |
| Logistic regression | 474659 | 25239 | 0.807568 | 0.197434 | 0.182116 | Higher education; Age; Sex Male; Ethnicity Other; Current smoking; Current drinking; Physical activity; BMI; Waist circumference; SBP; Antihypertensive; Triglycerides; HDL cholesterol; CRP |

Model performance was evaluated using five-fold stratified cross-validation in the prospective cohort.

Supplementary Table 11. NHANES 2011-2018 external validation of the education-prevalent T2D association.

| analysis | n | events | Effect (95% CI) | P value | covariates |
| --- | --- | --- | --- | --- | --- |
| prevalent T2D, weighted logistic model | 20502 | 2707 | 0.69 (0.61-0.78) | 2.16E-09 | age, sex, race/ethnicity, current smoking, BMI |

Supplementary Table 12. The heritability of all phenotypes.

| **Phenotype** | **Heritability (SE)** |
| --- | --- |
| BMI | 0.2114 (0.0021) |
| BMI (Male) | 0.1768 (0.0117) |
| BMI (Female) | 0.1707 (0.0091) |
| T1D | 0.0306 (0.0034) |
| T2D | 0.0532 (0.0034) |
| Educational attainment | 0.0753 (0.0021) |
| Cognitive performance | 0.1995 (0.0069) |
| Intelligence | 0.1894 (0.007) |

Supplementary Table 14. Causal estimates of educational attainment, cognitive performance and intelligence with BMI and diabetes using MR analyses.

| **Exposure** | **Outcome** | **Method** | **No. instruments** | **OR** | ***P*** |
| --- | --- | --- | --- | --- | --- |
| Educational attainment | BMI | IVW | 138 | 0.80 (0.74-0.86) | **4.65E-10** |
|  |  | MR Egger | 138 | 0.79 (0.58-1.07) | 0.127 |
|  |  | GSMR | 383 | 0.75 (0.74-0.76) | **1.24E-266** |
|  | BMI (Male) | IVW | 141 | 0.84 (0.77-0.91) | **0.0001** |
|  |  | MR Egger | 141 | 1.00 (0.69-1.45) | 0.999 |
|  |  | GSMR | 482 | 0.82 (0.79-0.86) | **3.10E-21** |
|  | BMI (Female) | IVW | 141 | 0.82 (0.75-0.89) | **4.83E-06** |
|  |  | MR Egger | 141 | 0.81 (0.56-1.17) | 0.259 |
|  |  | GSMR | 478 | 0.80 (0.77-0.83) | **7.63E-31** |
|  | T1D | IVW | 303 | 0.71 (0.52-0.96) | 0.0257 |
|  |  | MR Egger | 303 | 1.75 (0.53-5.75) | 0.358 |
|  |  | GSMR | 537 | 0.59 (0.53-0.66) | **3.96E-20** |
|  | T2D | IVW | 248 | 0.54 (0.47-0.61) | 1.49E-22 |
|  |  | MR Egger | 248 | 0.73 (0.42-1.28) | 0.804 |
|  |  | GSMR | 463 | 0.56 (0.53-0.60) | **2.09E-69** |
| Cognitive performance | BMI | IVW | 68 | 0.91 (0.83-0.99) | 0.0293 |
|  |  | MR Egger | 68 | 0.78 (0.53-1.15) | 0.207 |
|  |  | GSMR | 123 | 0.88 (0.87-0.90) | **1.35E-44** |
|  | BMI (Male) | IVW | 71 | 0.91 (0.82-1.00) | 0.048 |
|  |  | MR Egger | 71 | 0.64 (0.42-0.96) | 0.0344 |
|  |  | GSMR | 171 | 0.92 (0.89-0.96) | **0.000231** |
|  | BMI (Female) | IVW | 71 | 0.94 (0.85-1.03) | 0.194 |
|  |  | MR Egger | 71 | 0.77 (0.51-1.17) | 0.231 |
|  |  | GSMR | 169 | 0.90 (0.87-0.94) | **3.29E-07** |
|  | T1D | IVW | 139 | 0.99 (0.77-1.27) | 0.931 |
|  |  | MR Egger | 139 | 0.79 (0.27-2.35) | 0.674 |
|  |  | GSMR | 199 | 0.83 (0.74-0.93) | **0.00117** |
|  | T2D | IVW | 107 | 0.79 (0.69-0.91) | **0.0012** |
|  |  | MR Egger | 107 | 1.06 (0.53-2.13) | 0.864 |
|  |  | GSMR | 167 | 0.82 (0.77-0.88) | **1.55E-09** |
| Intelligence | BMI | IVW | 95 | 0.89 (0.83-0.96) | **0.00229** |
|  |  | MR Egger | 95 | 0.95 (0.67-1.35) | 0.773 |
|  |  | GSMR | 147 | 0.85 (0.84-0.87) | **5.43E-81** |
|  | BMI (Male) | IVW | 100 | 0.90 (0.83-0.98) | 0.0119 |
|  |  | MR Egger | 100 | 0.73 (0.50-1.07) | 0.111 |
|  |  | GSMR | 191 | 0.94 (0.91-0.98) | 0.0067 |
|  | BMI (Female) | IVW | 99 | 0.92 (0.84-1.00) | 0.0413 |
|  |  | MR Egger | 99 | 0.79 (0.53-1.17) | 0.242 |
|  |  | GSMR | 188 | 0.90 (0.86-0.93) | **6.64E-08** |
|  | T1D | IVW | 168 | 0.81 (0.68-0.95) | 0.0117 |
|  |  | MR Egger | 168 | 0.93 (0.42-2.06) | 0.863 |
|  |  | GSMR | 226 | 0.78 (0.70-0.87) | **6.85E-06** |
|  | T2D | IVW | 141 | 0.82 (0.72-0.93) | **0.0016** |
|  |  | MR Egger | 141 | 0.99 (0.52-1.90) | 0.984 |
|  |  | GSMR | 191 | 0.84 (0.79-0.89) | **4.00E-08** |

The statistically significant causality is defined to be *P*< 0.05/15 = 0.0033 after correcting for multiple testing.

Supplementary Table 15. MR instrument strength and leave-one-out IVW diagnostics.

| analysis | n_instruments | mean_F_statistic | leave_one_out_beta_min | leave_one_out_beta_max | OR_min | OR_max |
| --- | --- | --- | --- | --- | --- | --- |
| Educational attainment -> BMI | 141 | 48.23 | -0.2055 | -0.1602 | 0.8142 | 0.852 |
| Educational attainment -> T1D | 303 | 49.13 | -0.4545 | -0.259 | 0.6348 | 0.7719 |
| Educational attainment -> T2D | 248 | 50.06 | -0.6464 | -0.6085 | 0.5239 | 0.5442 |
| T2D -> Educational attainment | 117 | 71.27 | -0.01486 | -0.01161 | 0.9853 | 0.9885 |

Supplementary Table 16. Residual global and outlier diagnostics for key MR analyses.

| Analysis | N outliers | Outlier SNPs | Outlier-corrected OR (95% CI) | Corrected P |
| --- | --- | --- | --- | --- |
| Educational attainment -> BMI | 2 | rs1620977;rs9436866 | 0.82 (0.77-0.88) | 8.82E-08 |
| Educational attainment -> T1D | 6 | rs1689510;rs2256965;rs2347526;rs337637;rs4787457;rs74998289 | 0.64 (0.56-0.74) | 1.74E-09 |
| Educational attainment -> T2D | 5 | rs11601122;rs2256965;rs2725370;rs7029718;rs9914918 | 0.53 (0.49-0.58) | 1.99E-51 |
| T2D -> Educational attainment | 8 | rs10087241;rs12945601;rs13239186;rs1552224;rs7561798;rs7729395;rs853974;rs9894220 | 0.99 (0.98-0.99) | 2.64E-06 |

Supplementary Table 17. Approximate Steiger directionality filtering for key MR analyses.

| Analysis | N Steiger pass | N Steiger fail | Percent pass | Steiger-filtered OR (95% CI) | Steiger-filtered IVW P |
| --- | --- | --- | --- | --- | --- |
| Educational attainment -> BMI | 140 | 1 | 99.3 | 0.85 (0.79-0.91) | 7.63E-06 |
| Educational attainment -> T1D | 300 | 3 | 99 | 0.71 (0.61-0.82) | 1.80E-06 |
| Educational attainment -> T2D | 176 | 72 | 71 | 0.75 (0.68-0.82) | 2.31E-09 |
| T2D -> Educational attainment | 117 | 0 | 100 | 0.99 (0.98-0.99) | 5.16E-07 |

Supplementary Table 18. More stringent exposure P-value threshold sensitivity analyses for key MR analyses.

| Analysis | Instrument threshold | N instruments | IVW OR (95% CI) | IVW P | Weighted median OR | Weighted median P |
| --- | --- | --- | --- | --- | --- | --- |
| Educational attainment -> BMI | p_exposure < 5e-08 | 141 | 0.84 (0.79-0.90) | 1.47E-06 | 0.824 | 3.55E-06 |
| Educational attainment -> BMI | p_exposure < 1e-08 | 117 | 0.84 (0.78-0.90) | 2.61E-06 | 0.824 | 1.35E-04 |
| Educational attainment -> BMI | p_exposure < 5e-09 | 110 | 0.83 (0.77-0.90) | 2.44E-06 | 0.814 | 7.83E-05 |
| Educational attainment -> T1D | p_exposure < 5e-08 | 303 | 0.71 (0.61-0.81) | 1.21E-06 | 0.585 | 9.89E-13 |
| Educational attainment -> T1D | p_exposure < 1e-08 | 255 | 0.67 (0.58-0.78) | 1.33E-07 | 0.573 | 3.01E-18 |
| Educational attainment -> T1D | p_exposure < 5e-09 | 240 | 0.68 (0.59-0.79) | 6.73E-07 | 0.585 | 4.25E-14 |
| Educational attainment -> T2D | p_exposure < 5e-08 | 248 | 0.54 (0.49-0.58) | 6.97E-52 | 0.557 | 5.18E-16 |
| Educational attainment -> T2D | p_exposure < 1e-08 | 208 | 0.54 (0.49-0.59) | 1.56E-46 | 0.562 | 5.53E-19 |
| Educational attainment -> T2D | p_exposure < 5e-09 | 196 | 0.54 (0.50-0.59) | 8.29E-44 | 0.567 | 3.94E-18 |
| T2D -> Educational attainment | p_exposure < 5e-08 | 117 | 0.99 (0.98-0.99) | 5.16E-07 | 0.989 | 2.59E-07 |
| T2D -> Educational attainment | p_exposure < 1e-08 | 93 | 0.99 (0.98-0.99) | 1.20E-05 | 0.989 | 1.39E-06 |
| T2D -> Educational attainment | p_exposure < 5e-09 | 91 | 0.99 (0.98-0.99) | 1.66E-05 | 0.989 | 5.80E-06 |

Supplementary Table 19. Causal estimates of educational attainment, cognitive performance and intelligence with BMI and diabetes using multivariable MR analysis.

| **Exposure** | **Outcome** | **N_SNP_** | **OR (95%CI)** | ***P* value** |
| --- | --- | --- | --- | --- |
| EA | BMI | 123 | 0.85 (0.74-0.98) | **0.026** |
| CP |  | 49 | 1.32 (0.87-1.99) | 0.19 |
| INT |  | 50 | 0.70 (0.45-1.07) | 0.097 |
| EA | BMI (Male) | 124 | 0.93 (0.79-1.11) | 0.43 |
| CP |  | 51 | 1.22 (0.74-2.00) | 0.43 |
| INT |  | 52 | 0.70 (0.42-1.16) | 0.17 |
| EA | BMI (Female) | 124 | 0.83 (0.70-0.98) | **0.028** |
| CP |  | 51 | 1.03 (0.64-1.67) | 0.9 |
| INT |  | 52 | 0.93 (0.57-1.53) | 0.79 |
| EA | T1D | 258 | 0.88 (0.59-1.30) | 0.51 |
| CP |  | 86 | 0.90 (0.30-2.73) | 0.85 |
| INT |  | 83 | 0.95 (0.30-2.97) | 0.93 |
| EA | T2D | 218 | 0.54 (0.43-0.67) | **1.59E-08** |
| CP |  | 66 | 0.62 (0.32-1.19) | 0.15 |
| INT |  | 70 | 1.74 (0.89-3.38) | 0.10 |

The statistically significant causality is defined to be *P*< 0.05/5 = 0.01 after correcting for multiple testing.

Supplementary Table 20. Causal estimates of BMI and diabetes on educational attainment, cognitive performance and intelligence using MR analyses.

| **Exposure** | **Outcome** | **Method** | **No. instruments** | **OR** | ***P*** |
| --- | --- | --- | --- | --- | --- |
| BMI | Educational attainment | IVW | 422 | 0.85 (0.83-0.87) | **3.21E-24** |
|  |  | MR Egger | 422 | 0.96 (0.91-1.01) | 0.115 |
|  |  | GSMR | 1006 | 0.90 (0.89-0.91) | **1.33E-129** |
|  | Cognitive performance | IVW | 418 | 0.88 (0.85-0.92) | **5.11E-09** |
|  |  | MR Egger | 418 | 0.95 (0.86-1.06) | 0.383 |
|  |  | GSMR | 1042 | 0.99 (0.98-1.01) | 0.377 |
|  | Intelligence | IVW | 364 | 0.86 (0.82-0.89) | **4.68E-14** |
|  |  | MR Egger | 364 | 0.96 (0.86-1.06) | 0.422 |
|  |  | GSMR | 1053 | 0.93 (0.92-0.94) | **1.37E-26** |
| BMI (Male) | Educational attainment | IVW | 30 | 0.95 (0.90-1.01) | 0.0738 |
|  |  | MR Egger | 30 | 1.05 (0.90-1.23) | 0.506 |
|  |  | GSMR | 30 | 0.95 (0.93-0.97) | **1.71E-06** |
|  | Cognitive performance | IVW | 30 | 0.92 (0.83-1.01) | 0.0772 |
|  |  | MR Egger | 30 | 1.02 (0.79-1.33) | 0.856 |
|  |  | GSMR | 32 | 0.97 (0.94-1.00) | 0.0934 |
|  | Intelligence | IVW | 30 | 0.91 (0.82-1.00) | 0.0401 |
|  |  | MR Egger | 30 | 1.07 (0.83-1.37) | 0.629 |
|  |  | GSMR | 32 | 0.96 (0.93-0.99) | 0.0182 |
| BMI (Female) | Educational attainment | IVW | 37 | 0.94 (0.89-0.99) | 0.0241 |
|  |  | MR Egger | 37 | 1.11 (0.97-1.28) | 0.139 |
|  |  | GSMR | 39 | 0.94 (0.93-0.96) | **4.20E-10** |
|  | Cognitive performance | IVW | 337 | 0.95 (0.88-1.03) | 0.245 |
|  |  | MR Egger | 37 | 1.04 (0.84-1.29) | 0.728 |
|  |  | GSMR | 42 | 0.96 (0.94-0.99) | 0.0113 |
|  | Intelligence | IVW | 37 | 0.95 (0.88-1.03) | 0.222 |
|  |  | MR Egger | 37 | 1.09 (0.89-1.34) | 0.418 |
|  |  | GSMR | 41 | 0.96 (0.93-0.98) | **0.00181** |
| T1D | Educational attainment | IVW | 68 | 1.00 (1.00-1.01) | 0.596 |
|  |  | MR Egger | 68 | 1.00 (0.99-1.01) | 0.856 |
|  |  | GSMR | 223 | 1.00 (1.00-1.00) | 0.434 |
|  | Cognitive performance | IVW | 68 | 1.00 (1.00-1.01) | 0.336 |
|  |  | MR Egger | 68 | 1.01 (1.00-1.03) | 0.128 |
|  |  | GSMR | 209 | 1.00 (1.00-1.00) | 0.903 |
|  | Intelligence | IVW | 117 | 0.99 (0.98-1.01) | 0.213 |
|  |  | MR Egger | 117 | 1.02 (0.99-1.06) | 0.234 |
|  |  | GSMR | 235 | 1.00 (1.00-1.00) | 0.348 |
| T2D | Educational attainment | IVW | 117 | 0.99 (0.98-1.00) | 0.0195 |
|  |  | MR Egger | 117 | 1.01 (0.98-1.04) | 0.534 |
|  |  | GSMR | 123 | 0.99 (0.99-1.00) | **0.000426** |
|  | Cognitive performance | IVW | 117 | 1.00 (0.98-1.01) | 0.738 |
|  |  | MR Egger | 117 | 1.03 (0.99-1.07) | 0.0913 |
|  |  | GSMR | 127 | 0.99 (0.98-1.00) | 0.0390 |
|  | Intelligence | IVW | 117 | 0.99 (0.98-1.01) | 0.213 |
|  |  | MR Egger | 117 | 1.02 (0.99-1.06) | 0.234 |
|  |  | GSMR | 130 | 0.99 (0.98-1.00) | 0.0107 |

The statistically significant causality is defined to be *P* < 0.05/15 = 0.0033 after correcting for multiple testing.

Supplementary Table 21. Tissue-type-specific enrichment of SNP heritability for BMI estimated using S-LDSC.

| **Type** | **Coefficient** | **SE** | **P value** |
| --- | --- | --- | --- |
| A08.186.211.464.Limbic.System | 1.233E-08 | 2.26E-09 | 2.49E-08 |
| A08.186.211.730.885.287.500.Cerebral.Cortex | 1.146E-08 | 2.13E-09 | 3.91E-08 |
| A08.186.211.464.405.Hippocampus | 1.168E-08 | 2.19E-09 | 4.64E-08 |
| A08.186.211.Brain | 1.161E-08 | 2.39E-09 | 5.96E-07 |
| A08.186.211.464.710.225.Entorhinal.Cortex | 1.224E-08 | 2.56E-09 | 8.52E-07 |
| Brain_Frontal_Cortex_(BA9) | 1.019E-08 | 2.21E-09 | 2E-06 |
| A08.186.211.132.Brain.Stem | 9.764E-09 | 2.33E-09 | 1.4E-05 |
| A08.186.211.132.810.428.200.Cerebellum | 9.572E-09 | 2.33E-09 | 2E-05 |
| Brain_Putamen_(basal_ganglia) | 8.797E-09 | 2.18E-09 | 2.76E-05 |
| A08.186.211.730.885.287.500.270.Frontal.Lobe | 9.941E-09 | 2.48E-09 | 3.11E-05 |
| Brain_Nucleus_accumbens_(basal_ganglia) | 9.054E-09 | 2.31E-09 | 4.42E-05 |
| A08.186.211.865.428.Metencephalon | 8.831E-09 | 2.31E-09 | 6.64E-05 |
| Brain_Anterior_cingulate_cortex_(BA24) | 8.427E-09 | 2.23E-09 | 8.1E-05 |
| Brain_Caudate_(basal_ganglia) | 8.339E-09 | 2.27E-09 | 0.000119 |
| A09.371.729.Retina | 1.063E-08 | 2.91E-09 | 0.000127 |

Supplementary Table 22. Tissue-type-specific enrichment of SNP heritability for educational attainment estimated using S-LDSC.

| **Type** | **Coefficient** | **SE** | **P value** |
| --- | --- | --- | --- |
| A08.186.211.730.885.287.500.Cerebral.Cortex | 8.37E-09 | 1.36E-09 | 3.58E-10 |
| A08.186.211.464.Limbic.System | 8.52E-09 | 1.4E-09 | 5.43E-10 |
| A08.186.211.464.710.225.Entorhinal.Cortex | 8.64E-09 | 1.42E-09 | 6.1E-10 |
| A08.186.211.464.405.Hippocampus | 8.79E-09 | 1.48E-09 | 1.42E-09 |
| A08.186.211.Brain | 7.37E-09 | 1.37E-09 | 3.96E-08 |
| A08.186.211.730.885.287.500.571.735.Visual.Cortex | 7.27E-09 | 1.47E-09 | 3.87E-07 |
| A08.186.211.730.885.287.500.270.Frontal.Lobe | 7.29E-09 | 1.52E-09 | 8.34E-07 |
| Brain_Frontal_Cortex_(BA9) | 7.55E-09 | 1.59E-09 | 1.03E-06 |
| Brain_Hippocampus | 7.7E-09 | 1.62E-09 | 1.04E-06 |
| Brain_Anterior_cingulate_cortex_(BA24) | 7.14E-09 | 1.6E-09 | 4.04E-06 |
| A08.186.211.730.885.287.500.670.Parietal.Lobe | 6.2E-09 | 1.45E-09 | 9.79E-06 |
| Brain_Amygdala | 7.12E-09 | 1.67E-09 | 1.01E-05 |
| Brain_Cortex | 7.08E-09 | 1.67E-09 | 1.06E-05 |
| A08.186.211.132.Brain.Stem | 6.18E-09 | 1.51E-09 | 2.03E-05 |
| Brain_Cerebellar_Hemisphere | 6.55E-09 | 1.61E-09 | 2.42E-05 |
| Brain_Hypothalamus | 6.41E-09 | 1.61E-09 | 3.38E-05 |
| Brain_Putamen_(basal_ganglia) | 6.31E-09 | 1.61E-09 | 4.46E-05 |
| Brain_Caudate_(basal_ganglia) | 6.25E-09 | 1.63E-09 | 6.53E-05 |
| A08.186.211.132.810.428.200.Cerebellum | 5.84E-09 | 1.54E-09 | 7.14E-05 |
| Brain_Cerebellum | 5.83E-09 | 1.53E-09 | 7.19E-05 |
| Brain_Nucleus_accumbens_(basal_ganglia) | 6.21E-09 | 1.64E-09 | 7.65E-05 |
| A08.186.211.865.428.Metencephalon | 5.64E-09 | 1.5E-09 | 8.18E-05 |

Supplementary Table 23. Tissue-type-specific enrichment of SNP heritability for cognitive performance estimated using S-LDSC.

| **Type** | **Coefficient** | **SE** | **P value** |
| --- | --- | --- | --- |
| A08.186.211.464.405.Hippocampus | 1.74E-08 | 2.27E-09 | 1.06E-14 |
| A08.186.211.730.885.287.500.Cerebral.Cortex | 1.87E-08 | 2.45E-09 | 1.19E-14 |
| A08.186.211.464.710.225.Entorhinal.Cortex | 1.79E-08 | 2.35E-09 | 1.36E-14 |
| A08.186.211.464.Limbic.System | 1.79E-08 | 2.42E-09 | 7.29E-14 |
| Brain_Frontal_Cortex_(BA9) | 1.68E-08 | 2.55E-09 | 2.47E-11 |
| A08.186.211.730.885.287.500.270.Frontal.Lobe | 1.58E-08 | 2.51E-09 | 1.59E-10 |
| Brain_Cortex | 1.63E-08 | 2.59E-09 | 1.69E-10 |
| A08.186.211.Brain | 1.61E-08 | 2.56E-09 | 1.71E-10 |
| A08.186.211.730.885.287.500.571.735.Visual.Cortex | 1.53E-08 | 2.44E-09 | 1.71E-10 |
| Brain_Anterior_cingulate_cortex_(BA24) | 1.49E-08 | 2.59E-09 | 4.02E-09 |
| A08.186.211.132.810.428.200.Cerebellum | 1.22E-08 | 2.22E-09 | 1.78E-08 |
| A08.186.211.865.428.Metencephalon | 1.19E-08 | 2.25E-09 | 5.54E-08 |
| A08.186.211.132.Brain.Stem | 1.23E-08 | 2.35E-09 | 8.71E-08 |
| Brain_Nucleus_accumbens_(basal_ganglia) | 1.37E-08 | 2.62E-09 | 8.93E-08 |
| A08.186.211.730.885.287.500.670.Parietal.Lobe | 1.35E-08 | 2.61E-09 | 1.19E-07 |
| Brain_Hippocampus | 1.3E-08 | 2.58E-09 | 2.43E-07 |
| Brain_Amygdala | 1.28E-08 | 2.63E-09 | 5.45E-07 |
| Brain_Hypothalamus | 1.2E-08 | 2.54E-09 | 1.09E-06 |
| A11.872.653.Neural.Stem.Cells | 1.28E-08 | 2.76E-09 | 1.73E-06 |
| Brain_Caudate_(basal_ganglia) | 1.12E-08 | 2.56E-09 | 5.99E-06 |
| Brain_Putamen_(basal_ganglia) | 1.13E-08 | 2.6E-09 | 7.71E-06 |
| Brain_Substantia_nigra | 1.08E-08 | 2.58E-09 | 1.32E-05 |
| Brain_Cerebellum | 8.8E-09 | 2.2E-09 | 3.04E-05 |
| Brain_Cerebellar_Hemisphere | 8.6E-09 | 2.27E-09 | 7.83E-05 |
| A09.371.729.Retina | 8.92E-09 | 2.4E-09 | 9.81E-05 |
| A08.186.211.730.885.287.249.Basal.Ganglia | 8.65E-09 | 2.47E-09 | 0.000236 |
| A08.186.211.730.317.Diencephalon | 7.84E-09 | 2.24E-09 | 0.000237 |

Supplementary Table 24. Tissue-type-specific enrichment of SNP heritability for intelligence estimated using S-LDSC.

| **Tpye** | **Coefficient** | **SE** | **P value** |
| --- | --- | --- | --- |
| A08.186.211.730.885.287.500.Cerebral.Cortex | 1.78E-08 | 2.25E-09 | 1.18E-15 |
| A08.186.211.464.Limbic.System | 1.76E-08 | 2.24E-09 | 1.75E-15 |
| A08.186.211.464.405.Hippocampus | 1.64E-08 | 2.09E-09 | 2.33E-15 |
| A08.186.211.464.710.225.Entorhinal.Cortex | 1.7E-08 | 2.2E-09 | 5.7E-15 |
| Brain_Frontal_Cortex_(BA9) | 1.75E-08 | 2.39E-09 | 1E-13 |
| A08.186.211.Brain | 1.6E-08 | 2.33E-09 | 3.35E-12 |
| Brain_Cortex | 1.7E-08 | 2.5E-09 | 4.98E-12 |
| Brain_Anterior_cingulate_cortex_(BA24) | 1.59E-08 | 2.42E-09 | 2.34E-11 |
| A08.186.211.730.885.287.500.270.Frontal.Lobe | 1.49E-08 | 2.39E-09 | 1.99E-10 |
| A08.186.211.730.885.287.500.571.735.Visual.Cortex | 1.34E-08 | 2.32E-09 | 3.97E-09 |
| A08.186.211.132.810.428.200.Cerebellum | 1.3E-08 | 2.25E-09 | 4.05E-09 |
| A08.186.211.865.428.Metencephalon | 1.3E-08 | 2.26E-09 | 4.24E-09 |
| Brain_Amygdala | 1.41E-08 | 2.54E-09 | 1.3E-08 |
| A08.186.211.730.885.287.500.670.Parietal.Lobe | 1.31E-08 | 2.37E-09 | 1.51E-08 |
| A08.186.211.132.Brain.Stem | 1.26E-08 | 2.32E-09 | 2.63E-08 |
| Brain_Nucleus_accumbens_(basal_ganglia) | 1.3E-08 | 2.41E-09 | 3.51E-08 |
| Brain_Hippocampus | 1.36E-08 | 2.53E-09 | 4.16E-08 |
| A11.872.653.Neural.Stem.Cells | 1.39E-08 | 2.74E-09 | 2.02E-07 |
| Brain_Hypothalamus | 1.23E-08 | 2.45E-09 | 2.28E-07 |
| Brain_Cerebellar_Hemisphere | 1.03E-08 | 2.18E-09 | 1.09E-06 |
| A09.371.729.Retina | 9.88E-09 | 2.16E-09 | 2.5E-06 |
| Brain_Caudate_(basal_ganglia) | 1.11E-08 | 2.44E-09 | 2.83E-06 |
| Brain_Cerebellum | 9.18E-09 | 2.02E-09 | 2.84E-06 |
| Brain_Putamen_(basal_ganglia) | 1.13E-08 | 2.49E-09 | 2.96E-06 |
| Brain_Substantia_nigra | 1.09E-08 | 2.56E-09 | 1.1E-05 |

Supplementary Table 25. Cell-type-specific enrichment of SNP heritability using S-LDSC.

| **Traits** | **Name** | **Coefficient** | **SE** | **P** |
| --- | --- | --- | --- | --- |
| BMI | Neuron | 4.49E-09 | 2.48E-09 | **0.035** |
|  | Oligodendrocyte | 8.07E-10 | 2.57E-09 | 0.38 |
|  | Astrocyte | -4.04E-09 | 2.09E-09 | 0.97 |
| EA | Neuron | 5.96E-09 | 1.75E-09 | **0.00034** |
|  | Oligodendrocyte | 5.76E-10 | 1.70E-09 | 0.37 |
|  | Astrocyte | -2.23E-09 | 1.59E-09 | 0.92 |
| CP | Neuron | 1.25E-08 | 2.44E-09 | **1.40E-07** |
|  | Oligodendrocyte | -2.58E-09 | 2.60E-09 | 0.84 |
|  | Astrocyte | -3.09E-09 | 2.32E-09 | 0.91 |
| INT | Neuron | 1.20E-08 | 2.46E-09 | **4.90E-07** |
|  | Oligodendrocyte | -1.35E-09 | 2.42E-09 | 0.71 |
|  | Astrocyte | -3.01E-09 | 2.26E-09 | 0.91 |
| T1D | Neuron | 2.08E-09 | 2.86E-09 | 0.23 |
|  | Oligodendrocyte | 1.15E-10 | 1.98E-09 | 0.48 |
|  | Astrocyte | -2.30E-09 | 1.69E-09 | 0.91 |
| T2D | Neuron | 1.92E-09 | 1.75E-09 | 0.14 |
|  | Oligodendrocyte | 1.04E-09 | 1.27E-09 | 0.21 |
|  | Astrocyte | -2.14E-09 | 1.29E-09 | 0.95 |

Supplementary Table 26. Tissue-type-specific enrichment of SNP heritability for T1D estimated using S-LDSC.

| **Type** | **Coefficient** | **SE** | **P value** |
| --- | --- | --- | --- |
| A10.549.Lymphoid.Tissue | 8.81E-09 | 1.81E-09 | 5.62E-07 |
| A10.549.400.Lymph.Nodes | 7.61E-09 | 1.84E-09 | 1.7E-05 |
| Spleen | 7.41E-09 | 1.82E-09 | 2.23E-05 |
| A02.835.583.443.800.800.Synovial.Fluid | 7.99E-09 | 1.97E-09 | 2.53E-05 |
| A11.118.637.555.567.569.T.Lymphocytes | 9.09E-09 | 2.27E-09 | 3.16E-05 |
| A15.382.520.604.700.Spleen | 6.4E-09 | 1.62E-09 | 3.79E-05 |
| A15.145.229.Blood.Cells | 8.95E-09 | 2.33E-09 | 6.36E-05 |
| A15.382.490.555.567.537.Killer.Cells..Natural | 7.11E-09 | 1.97E-09 | 0.00016 |
| A11.118.637.555.567.569.200.700.T.Lymphocytes..Regulatory | 6.92E-09 | 1.96E-09 | 0.000213 |

Supplementary Table 27. Tissue-type-specific enrichment of SNP heritability for T2D estimated using S-LDSC.

| **Type** | **Coefficient** | **SE** | **P value** |
| --- | --- | --- | --- |
| Pancreas | 3.36E-09 | 9.85E-10 | 0.0003 |
| Liver | 2.44E-09 | 1.18E-09 | 0.0196 |
| A08.186.211.464.Limbic.System | 1.79E-09 | 9.38E-10 | 0.0283 |
| A08.186.211.730.885.287.500.Cerebral.Cortex | 1.68E-09 | 9.14E-10 | 0.0327 |
| A11.436.348.Hepatocytes | 4.62E-09 | 2.51E-09 | 0.0328 |
| Uterus | 3.72E-09 | 2.08E-09 | 0.0367 |
| A11.118.637.555.567.562.B.Lymphocytes | 2.17E-09 | 1.23E-09 | 0.0392 |

Supplementary Table 28. Functional genes that may be causally associated with education phenotypes, obesity phenotypes and diabetes phenotypes using SMR.

| **Phenotype** | **probeID** | **Chr** | **Gene** | **b_SMR** | **se_SMR** | **p_SMR** | **p_HEIDI** | **nsnp_HEIDI** |
| --- | --- | --- | --- | --- | --- | --- | --- | --- |
| BMI | ENSG00000268575 | 1 | *RP1-283E3.8* | -0.032 | 0.005 | 2.29E-10 | 0.44 | 18 |
|  | ENSG00000008130 | 1 | *NADK* | -0.040 | 0.007 | 1.76E-08 | 0.41 | 16 |
|  | ENSG00000226849 | 1 | *RP4-635E18.7* | 0.026 | 0.006 | 3.50E-06 | 0.34 | 15 |
|  | ENSG00000116771 | 1 | *AGMAT* | -0.022 | 0.005 | 1.54E-06 | 0.97 | 17 |
|  | ENSG00000160094 | 1 | *ZNF362* | -0.046 | 0.010 | 2.23E-06 | 0.06 | 16 |
|  | ENSG00000172260 | 1 | *NEGR1* | 0.069 | 0.009 | 1.75E-13 | 0.19 | 20 |
|  | ENSG00000162616 | 1 | *DNAJB4* | -0.034 | 0.007 | 1.46E-07 | 0.09 | 20 |
|  | ENSG00000198890 | 1 | *PRMT6* | -0.026 | 0.005 | 7.11E-09 | 0.44 | 9 |
|  | ENSG00000162877 | 1 | *PM20D1* | 0.019 | 0.004 | 2.46E-06 | 0.97 | 20 |
|  | ENSG00000138031 | 2 | *ADCY3* | -0.075 | 0.009 | 1.61E-18 | 0.11 | 20 |
|  | ENSG00000171303 | 2 | *KCNK3* | -0.041 | 0.006 | 1.93E-12 | 0.32 | 20 |
|  | ENSG00000213639 | 2 | *PPP1CB* | -0.051 | 0.010 | 2.13E-07 | 0.18 | 20 |
|  | ENSG00000071967 | 2 | *CYBRD1* | 0.021 | 0.004 | 2.94E-08 | 0.16 | 20 |
|  | ENSG00000157036 | 3 | *EXOG* | -0.020 | 0.004 | 4.58E-06 | 0.43 | 20 |
|  | ENSG00000246375 | 4 | *RP11-10L7.1* | 0.009 | 0.002 | 2.95E-08 | 0.07 | 20 |
|  | ENSG00000249244 | 4 | *RP11-548H18.2* | 0.014 | 0.003 | 1.86E-06 | 0.22 | 20 |
|  | ENSG00000055147 | 5 | *FAM114A2* | 0.035 | 0.006 | 1.86E-08 | 0.08 | 20 |
|  | ENSG00000112308 | 6 | *C6orf62* | -0.018 | 0.004 | 6.56E-07 | 0.06 | 20 |
|  | ENSG00000272462 | 6 | *U91328.19* | 0.017 | 0.003 | 1.05E-06 | 0.26 | 20 |
|  | ENSG00000135547 | 6 | *HEY2* | -0.024 | 0.004 | 1.51E-08 | 0.08 | 20 |
|  | ENSG00000198945 | 6 | *L3MBTL3* | 0.023 | 0.004 | 2.51E-07 | 0.09 | 20 |
|  | ENSG00000188073 | 7 | *PMS2P10* | -0.036 | 0.007 | 1.13E-07 | 0.05 | 5 |
|  | ENSG00000186088 | 7 | *PION* | -0.017 | 0.003 | 1.72E-09 | 0.15 | 19 |
|  | ENSG00000106367 | 7 | *AP1S1* | 0.009 | 0.002 | 4.37E-09 | 0.06 | 20 |
|  | ENSG00000253893 | 8 | *FAM85B* | -0.024 | 0.005 | 4.08E-07 | 0.31 | 20 |
|  | ENSG00000255310 | 8 | *AF131215.2* | 0.053 | 0.011 | 1.60E-06 | 0.16 | 20 |
|  | ENSG00000254423 | 8 | *RP11-351I21.7* | 0.024 | 0.005 | 4.42E-06 | 0.40 | 20 |
|  | ENSG00000172728 | 8 | *FUT10* | -0.025 | 0.005 | 2.19E-06 | 0.80 | 20 |
|  | ENSG00000180921 | 8 | *FAM83H* | -0.018 | 0.004 | 1.70E-06 | 0.07 | 20 |
|  | ENSG00000107341 | 9 | *UBE2R2* | 0.036 | 0.007 | 9.94E-08 | 0.07 | 11 |
|  | ENSG00000198467 | 9 | *TPM2* | -0.008 | 0.002 | 7.00E-06 | 0.09 | 20 |
|  | ENSG00000136875 | 9 | *PRPF4* | 0.016 | 0.003 | 5.41E-06 | 0.51 | 20 |
|  | ENSG00000138175 | 10 | *ARL3* | -0.048 | 0.010 | 2.11E-06 | 0.06 | 20 |
|  | ENSG00000166199 | 11 | *ALKBH3* | 0.029 | 0.005 | 3.27E-08 | 0.27 | 10 |
|  | ENSG00000175220 | 11 | *ARHGAP1* | -0.052 | 0.012 | 6.27E-06 | 0.55 | 3 |
|  | ENSG00000182450 | 11 | *KCNK4* | -0.043 | 0.008 | 1.04E-07 | 0.14 | 20 |
|  | ENSG00000126432 | 11 | *PRDX5* | 0.028 | 0.005 | 4.43E-09 | 0.17 | 20 |
|  | ENSG00000197345 | 11 | *MRPL21* | -0.008 | 0.002 | 5.28E-07 | 0.13 | 20 |
|  | ENSG00000132740 | 11 | *IGHMBP2* | 0.014 | 0.003 | 3.69E-06 | 0.11 | 20 |
|  | ENSG00000271751 | 11 | *RP11-110I1.14* | -0.030 | 0.006 | 2.45E-07 | 0.30 | 8 |
|  | ENSG00000160695 | 11 | *VPS11* | -0.024 | 0.004 | 8.78E-11 | 0.42 | 9 |
|  | ENSG00000176422 | 12 | *SPRYD4* | -0.034 | 0.007 | 1.73E-06 | 0.30 | 11 |
|  | ENSG00000130779 | 12 | *CLIP1* | -0.058 | 0.011 | 1.12E-07 | 0.10 | 14 |
|  | ENSG00000151327 | 14 | *FAM177A1* | 0.023 | 0.005 | 1.72E-06 | 0.25 | 20 |
|  | ENSG00000131323 | 14 | *TRAF3* | 0.059 | 0.011 | 4.69E-08 | 0.11 | 18 |
|  | ENSG00000075413 | 14 | *MARK3* | -0.035 | 0.006 | 3.74E-09 | 0.23 | 18 |
|  | ENSG00000256053 | 14 | *APOPT1* | -0.035 | 0.006 | 4.47E-09 | 0.23 | 20 |
|  | ENSG00000169783 | 15 | *LINGO1* | 0.024 | 0.005 | 4.59E-06 | 0.06 | 20 |
|  | ENSG00000185615 | 16 | *PDIA2* | -0.045 | 0.009 | 3.66E-07 | 0.60 | 12 |
|  | ENSG00000262185 | 16 | *RP11-462G12.1* | -0.053 | 0.008 | 1.70E-10 | 0.53 | 8 |
|  | ENSG00000184110 | 16 | *EIF3C* | 0.048 | 0.005 | 8.87E-19 | 0.29 | 20 |
|  | ENSG00000251417 | 16 | *RP11-1348G14.4* | -0.050 | 0.009 | 2.04E-08 | 0.08 | 20 |
|  | ENSG00000176953 | 16 | *NFATC2IP* | 0.108 | 0.019 | 6.11E-09 | 0.40 | 16 |
|  | ENSG00000174938 | 16 | *SEZ6L2* | -0.044 | 0.009 | 2.51E-06 | 0.72 | 7 |
|  | ENSG00000174939 | 16 | *ASPHD1* | 0.077 | 0.012 | 5.63E-10 | 0.06 | 17 |
|  | ENSG00000169900 | 16 | *PYDC1* | 0.050 | 0.010 | 5.94E-07 | 0.84 | 12 |
|  | ENSG00000141076 | 16 | *CIRH1A* | 0.020 | 0.004 | 6.64E-08 | 0.06 | 4 |
|  | ENSG00000090861 | 16 | *AARS* | 0.044 | 0.008 | 1.56E-08 | 0.19 | 16 |
|  | ENSG00000141503 | 17 | *MINK1* | 0.041 | 0.008 | 2.86E-07 | 0.16 | 20 |
|  | ENSG00000161929 | 17 | *SCIMP* | 0.016 | 0.004 | 6.36E-06 | 0.08 | 20 |
|  | ENSG00000129197 | 17 | *RPAIN* | -0.048 | 0.010 | 4.13E-07 | 0.43 | 20 |
|  | ENSG00000170425 | 17 | *ADORA2B* | -0.023 | 0.004 | 4.04E-09 | 0.64 | 18 |
|  | ENSG00000005955 | 17 | *GGNBP2* | -0.033 | 0.005 | 1.58E-11 | 0.06 | 6 |
|  | ENSG00000171634 | 17 | *BPTF* | -0.034 | 0.006 | 1.05E-07 | 0.61 | 12 |
|  | ENSG00000186665 | 17 | *C17orf58* | 0.037 | 0.007 | 1.48E-07 | 0.14 | 7 |
|  | ENSG00000132481 | 17 | *TRIM47* | -0.025 | 0.005 | 7.57E-07 | 0.74 | 20 |
|  | ENSG00000169689 | 17 | *STRA13* | -0.009 | 0.002 | 1.64E-06 | 0.15 | 16 |
|  | ENSG00000141452 | 18 | *C18orf8* | -0.042 | 0.005 | 1.63E-17 | 0.83 | 20 |
|  | ENSG00000141458 | 18 | *NPC1* | -0.035 | 0.005 | 1.90E-13 | 0.17 | 20 |
|  | ENSG00000105229 | 19 | *PIAS4* | -0.063 | 0.012 | 3.82E-07 | 0.26 | 13 |
|  | ENSG00000268595 | 19 | *CTD-3187F8.2* | -0.013 | 0.003 | 1.34E-06 | 0.12 | 9 |
|  | ENSG00000197586 | 20 | *ENTPD6* | 0.040 | 0.008 | 2.29E-06 | 0.26 | 9 |
|  | ENSG00000125510 | 20 | *OPRL1* | 0.033 | 0.006 | 3.64E-07 | 0.32 | 9 |
| EA | ENSG00000116406 | 1 | *EDEM3* | 0.016 | 0.004 | 2.96E-06 | 0.79 | 20 |
|  | ENSG00000171103 | 2 | *TRMT61B* | 0.014 | 0.003 | 2.78E-06 | 0.64 | 20 |
|  | ENSG00000135976 | 2 | *ANKRD36* | -0.018 | 0.004 | 3.65E-06 | 0.08 | 20 |
|  | ENSG00000228389 | 2 | *AC068039.4* | 0.009 | 0.002 | 6.76E-08 | 0.11 | 20 |
|  | ENSG00000243477 | 3 | *NAT6* | -0.031 | 0.006 | 3.04E-07 | 0.10 | 20 |
|  | ENSG00000180376 | 3 | *CCDC66* | 0.015 | 0.003 | 3.41E-06 | 0.17 | 20 |
|  | ENSG00000239519 | 3 | *CADM2-AS1* | 0.018 | 0.004 | 2.94E-06 | 0.12 | 20 |
|  | ENSG00000125388 | 4 | *GRK4* | -0.021 | 0.003 | 9.85E-10 | 0.05 | 20 |
|  | ENSG00000228716 | 5 | *DHFR* | 0.007 | 0.002 | 6.46E-06 | 0.07 | 20 |
|  | ENSG00000172201 | 6 | *ID4* | -0.049 | 0.010 | 2.55E-06 | 0.75 | 20 |
|  | ENSG00000187837 | 6 | *HIST1H1C* | 0.024 | 0.005 | 3.51E-06 | 0.05 | 20 |
|  | ENSG00000078319 | 7 | *PMS2P1* | 0.031 | 0.007 | 2.16E-06 | 0.22 | 20 |
|  | ENSG00000078487 | 7 | *ZCWPW1* | -0.020 | 0.004 | 1.95E-08 | 0.32 | 20 |
|  | ENSG00000176209 | 8 | *C8orf40* | -0.010 | 0.002 | 1.78E-09 | 0.05 | 20 |
|  | ENSG00000254081 | 8 | *CTD-3025N20.2* | 0.015 | 0.003 | 6.20E-06 | 0.26 | 20 |
|  | ENSG00000253741 | 8 | *CTD-2292P10.4* | 0.012 | 0.003 | 1.83E-06 | 0.22 | 20 |
|  | ENSG00000167702 | 8 | *KIFC2* | -0.038 | 0.008 | 8.15E-07 | 0.07 | 20 |
|  | ENSG00000081377 | 9 | *CDC14B* | -0.016 | 0.003 | 1.92E-06 | 0.08 | 20 |
|  | ENSG00000119328 | 9 | *FAM206A* | -0.031 | 0.006 | 8.30E-08 | 0.43 | 20 |
|  | ENSG00000095485 | 10 | *CWF19L1* | 0.007 | 0.002 | 5.38E-06 | 0.12 | 20 |
|  | ENSG00000172500 | 11 | *FIBP* | -0.024 | 0.005 | 1.95E-06 | 0.09 | 20 |
|  | ENSG00000256885 | 11 | *AP001877.1* | 0.025 | 0.005 | 2.68E-08 | 0.40 | 20 |
|  | ENSG00000167550 | 12 | *RHEBL1* | 0.038 | 0.008 | 3.35E-07 | 0.36 | 20 |
|  | ENSG00000188599 | 16 | *NPIPP1* | -0.030 | 0.006 | 2.99E-06 | 0.10 | 12 |
|  | ENSG00000176046 | 16 | *NUPR1* | -0.059 | 0.012 | 1.77E-06 | 0.62 | 20 |
|  | ENSG00000197165 | 16 | *SULT1A2* | -0.024 | 0.005 | 1.19E-07 | 0.07 | 20 |
|  | ENSG00000184110 | 16 | *EIF3C* | -0.029 | 0.004 | 1.34E-12 | 0.28 | 20 |
|  | ENSG00000251417 | 16 | *RP11-1348G14.4* | 0.030 | 0.006 | 8.88E-07 | 0.18 | 20 |
|  | ENSG00000176953 | 16 | *NFATC2IP* | -0.065 | 0.012 | 1.69E-07 | 0.57 | 20 |
|  | ENSG00000103479 | 16 | *RBL2* | -0.008 | 0.002 | 1.01E-06 | 0.10 | 20 |
|  | ENSG00000170175 | 17 | *CHRNB1* | 0.032 | 0.006 | 3.23E-07 | 0.07 | 20 |
|  | ENSG00000225190 | 17 | *PLEKHM1* | 0.040 | 0.009 | 2.46E-06 | 0.84 | 7 |
|  | ENSG00000131484 | 17 | *RP11-798G7.5* | 0.026 | 0.005 | 8.26E-07 | 0.95 | 11 |
|  | ENSG00000264070 | 17 | *DND1P1* | -0.025 | 0.005 | 3.21E-07 | 0.59 | 9 |
|  | ENSG00000262539 | 17 | *RP11-259G18.3* | -0.012 | 0.002 | 6.65E-11 | 0.08 | 20 |
|  | ENSG00000261575 | 17 | *RP11-259G18.1* | -0.022 | 0.004 | 8.44E-09 | 0.29 | 20 |
|  | ENSG00000159199 | 17 | *ATP5G1* | -0.022 | 0.004 | 5.34E-07 | 0.51 | 20 |
|  | ENSG00000159210 | 17 | *SNF8* | -0.019 | 0.004 | 1.90E-07 | 0.19 | 20 |
|  | ENSG00000141452 | 18 | *C18orf8* | 0.031 | 0.005 | 2.04E-12 | 0.10 | 20 |
|  | ENSG00000141458 | 18 | *NPC1* | 0.026 | 0.004 | 2.19E-10 | 0.57 | 20 |
|  | ENSG00000101109 | 20 | *STK4* | 0.026 | 0.005 | 8.54E-07 | 0.22 | 20 |
|  | ENSG00000101017 | 20 | *CD40* | 0.026 | 0.006 | 3.31E-06 | 0.18 | 20 |
|  | ENSG00000189306 | 22 | *RRP7A* | 0.019 | 0.004 | 5.92E-06 | 0.46 | 20 |
| CP | ENSG00000162517 | 1 | *PEF1* | 0.064 | 0.014 | 5.23E-06 | 0.20 | 20 |
|  | ENSG00000163016 | 2 | *ALMS1P* | -0.029 | 0.006 | 1.10E-06 | 0.26 | 20 |
|  | ENSG00000115526 | 2 | *CHST10* | 0.055 | 0.010 | 1.22E-07 | 0.07 | 20 |
|  | ENSG00000163938 | 3 | *GNL3* | -0.027 | 0.005 | 3.70E-07 | 0.06 | 20 |
|  | ENSG00000204963 | 5 | *PCDHA7* | -0.030 | 0.006 | 3.77E-06 | 0.26 | 20 |
|  | ENSG00000081377 | 9 | *CDC14B* | -0.032 | 0.006 | 4.13E-08 | 0.13 | 20 |
|  | ENSG00000095321 | 9 | *CRAT* | -0.042 | 0.009 | 7.29E-07 | 0.52 | 20 |
|  | ENSG00000172247 | 11 | *C1QTNF4* | 0.054 | 0.012 | 3.19E-06 | 0.66 | 20 |
|  | ENSG00000256885 | 11 | *AP001877.1* | 0.033 | 0.007 | 7.50E-07 | 0.95 | 20 |
|  | ENSG00000167550 | 12 | *RHEBL1* | 0.066 | 0.013 | 2.01E-07 | 0.14 | 20 |
|  | ENSG00000139531 | 12 | *SUOX* | -0.028 | 0.006 | 7.99E-07 | 0.24 | 20 |
|  | ENSG00000197728 | 12 | *RPS26* | 0.031 | 0.006 | 2.58E-08 | 0.33 | 20 |
|  | ENSG00000140323 | 15 | *DISP2* | -0.044 | 0.010 | 4.03E-06 | 0.44 | 12 |
|  | ENSG00000104112 | 15 | *SCG3* | -0.040 | 0.008 | 9.03E-07 | 0.12 | 20 |
|  | ENSG00000103723 | 15 | *AP3B2* | 0.040 | 0.008 | 1.55E-06 | 0.27 | 20 |
|  | ENSG00000184110 | 16 | *EIF3C* | -0.046 | 0.007 | 1.06E-11 | 0.44 | 20 |
|  | ENSG00000251417 | 16 | *RP11-1348G14.4* | 0.047 | 0.010 | 1.50E-06 | 0.13 | 20 |
|  | ENSG00000176953 | 16 | *NFATC2IP* | -0.098 | 0.020 | 4.38E-07 | 0.75 | 20 |
|  | ENSG00000103043 | 16 | *VAC14* | -0.059 | 0.012 | 8.35E-07 | 0.11 | 20 |
|  | ENSG00000005955 | 17 | *GGNBP2* | 0.039 | 0.007 | 5.18E-08 | 0.79 | 11 |
|  | ENSG00000131484 | 17 | *RP11-798G7.5* | 0.035 | 0.008 | 5.28E-06 | 0.42 | 11 |
|  | ENSG00000264070 | 17 | *DND1P1* | -0.034 | 0.007 | 2.24E-06 | 0.23 | 9 |
|  | ENSG00000261575 | 17 | *RP11-259G18.1* | -0.028 | 0.006 | 3.21E-07 | 0.13 | 20 |
|  | ENSG00000202077 | 17 | *RNU1-60P* | -0.019 | 0.004 | 1.35E-06 | 0.45 | 20 |
|  | ENSG00000124207 | 20 | *CSE1L* | -0.120 | 0.023 | 2.72E-07 | 0.30 | 20 |
|  | ENSG00000100106 | 22 | *TRIOBP* | 0.037 | 0.007 | 5.32E-07 | 0.28 | 20 |
|  | ENSG00000128268 | 22 | *MGAT3* | 0.069 | 0.015 | 1.67E-06 | 0.46 | 20 |
|  | ENSG00000183066 | 22 | *WBP2NL* | -0.028 | 0.005 | 5.50E-09 | 0.09 | 20 |
| INT | ENSG00000163016 | 2 | *ALMS1P* | -0.027 | 0.006 | 2.95E-06 | 0.07 | 20 |
|  | ENSG00000196653 | 3 | *ZNF502* | 0.013 | 0.003 | 4.62E-06 | 0.09 | 20 |
|  | ENSG00000146007 | 5 | *ZMAT2* | -0.047 | 0.010 | 3.17E-06 | 0.40 | 20 |
|  | ENSG00000204963 | 5 | *PCDHA7* | -0.031 | 0.006 | 6.55E-07 | 0.20 | 20 |
|  | ENSG00000204962 | 5 | *PCDHA8* | -0.035 | 0.007 | 2.69E-06 | 0.10 | 20 |
|  | ENSG00000254081 | 8 | *CTD-3025N20.2* | 0.025 | 0.006 | 5.75E-06 | 0.32 | 20 |
|  | ENSG00000081377 | 9 | *CDC14B* | -0.028 | 0.006 | 5.58E-07 | 0.83 | 20 |
|  | ENSG00000095321 | 9 | *CRAT* | -0.040 | 0.008 | 9.79E-07 | 0.10 | 20 |
|  | ENSG00000256885 | 11 | *AP001877.1* | 0.028 | 0.006 | 6.64E-06 | 0.97 | 20 |
|  | ENSG00000167550 | 12 | *RHEBL1* | 0.058 | 0.012 | 6.69E-07 | 0.34 | 20 |
|  | ENSG00000197728 | 12 | *RPS26* | 0.026 | 0.005 | 9.40E-07 | 0.23 | 20 |
|  | ENSG00000140323 | 15 | *DISP2* | -0.042 | 0.009 | 6.41E-06 | 0.53 | 11 |
|  | ENSG00000103723 | 15 | *AP3B2* | 0.037 | 0.008 | 3.78E-06 | 0.42 | 20 |
|  | ENSG00000184110 | 16 | *EIF3C* | -0.051 | 0.007 | 1.61E-13 | 0.14 | 20 |
|  | ENSG00000251417 | 16 | *RP11-1348G14.4* | 0.049 | 0.010 | 6.85E-07 | 0.08 | 20 |
|  | ENSG00000176953 | 16 | *NFATC2IP* | -0.109 | 0.021 | 1.34E-07 | 0.81 | 20 |
|  | ENSG00000141140 | 17 | *MYO19* | -0.043 | 0.009 | 3.90E-07 | 0.07 | 12 |
|  | ENSG00000005955 | 17 | *GGNBP2* | 0.036 | 0.007 | 1.57E-07 | 0.61 | 11 |
|  | ENSG00000202077 | 17 | *RNU1-60P* | -0.022 | 0.004 | 3.20E-08 | 0.47 | 20 |
|  | ENSG00000185658 | 21 | *BRWD1* | -0.058 | 0.013 | 5.28E-06 | 0.27 | 20 |
|  | ENSG00000100106 | 22 | *TRIOBP* | 0.034 | 0.007 | 6.14E-07 | 0.32 | 20 |
|  | ENSG00000128268 | 22 | *MGAT3* | 0.063 | 0.013 | 2.38E-06 | 0.25 | 20 |
|  | ENSG00000183066 | 22 | *WBP2NL* | -0.025 | 0.005 | 1.19E-07 | 0.47 | 20 |
| T1D | ENSG00000123685 | 1 | *BATF3* | 0.149 | 0.033 | 5.86E-06 | 0.58 | 20 |
|  | ENSG00000272273 | 6 | *XXbac-BPG252P9.10* | 0.430 | 0.077 | 2.4E-08 | 0.15 | 6 |
|  | ENSG00000137331 | 6 | *IER3* | 0.369 | 0.067 | 4.53E-08 | 0.07 | 5 |
|  | ENSG00000204616 | 6 | *TRIM31* | -0.172 | 0.032 | 8.28E-08 | 0.12 | 13 |
|  | ENSG00000204623 | 6 | *ZNRD1-AS1* | 0.336 | 0.066 | 2.93E-07 | 0.15 | 16 |
|  | ENSG00000005020 | 7 | *SKAP2* | 0.268 | 0.045 | 2.99E-09 | 0.69 | 20 |
|  | ENSG00000001626 | 7 | *CFTR* | -0.352 | 0.071 | 8E-07 | 0.22 | 18 |
|  | ENSG00000173457 | 11 | *PPP1R14B* | 0.242 | 0.049 | 9.32E-07 | 0.33 | 20 |
|  | ENSG00000270018 | 12 | *RP3-462E2.5* | 0.257 | 0.056 | 5.25E-06 | 0.13 | 17 |
|  | ENSG00000050820 | 16 | *BCAR1* | 0.445 | 0.093 | 1.84E-06 | 0.06 | 20 |
|  | ENSG00000197165 | 16 | *SULT1A2* | -0.148 | 0.031 | 2.06E-06 | 0.05 | 20 |
|  | ENSG00000214425 | 17 | *LRRC37A4P* | 0.068 | 0.013 | 1.75E-07 | 0.06 | 20 |
|  | ENSG00000267198 | 17 | *RP11-798G7.6* | -0.145 | 0.031 | 4.1E-06 | 0.20 | 20 |
|  | ENSG00000176909 | 19 | *MAMSTR* | 0.29 | 0.048 | 1.58E-09 | 0.11 | 20 |
| T2D | ENSG00000242142 | 3 | *SERBP1P3* | 0.040 | 0.009 | 3.23E-06 | 0.54 | 20 |
|  | ENSG00000163933 | 3 | *RFT1* | -0.089 | 0.019 | 3.29E-06 | 0.37 | 20 |
|  | ENSG00000230069 | 4 | *LRRC37A15P* | 0.093 | 0.018 | 1.46E-07 | 0.42 | 20 |
|  | ENSG00000248971 | 4 | *KRT8P46* | 0.105 | 0.021 | 6.43E-07 | 0.87 | 20 |
|  | ENSG00000223534 | 6 | *HLA-DQB1-AS1* | -0.086 | 0.014 | 2.92E-10 | 0.35 | 11 |
|  | ENSG00000179344 | 6 | *HLA-DQB1* | -0.081 | 0.013 | 4.01E-10 | 0.37 | 13 |
|  | ENSG00000232629 | 6 | *HLA-DQB2* | 0.109 | 0.021 | 2.18E-07 | 0.94 | 3 |
|  | ENSG00000196378 | 8 | *ZNF34* | 0.080 | 0.017 | 2.09E-06 | 0.77 | 12 |
|  | ENSG00000161016 | 8 | *RPL8* | -0.065 | 0.014 | 6.31E-06 | 0.68 | 14 |
|  | ENSG00000187486 | 11 | *KCNJ11* | 0.183 | 0.038 | 1.53E-06 | 0.35 | 16 |
|  | ENSG00000169783 | 15 | *LINGO1* | 0.102 | 0.022 | 5.72E-06 | 0.27 | 20 |
|  | ENSG00000159210 | 17 | *SNF8* | 0.096 | 0.017 | 1.01E-08 | 0.08 | 20 |
|  | ENSG00000159199 | 17 | *ATP5G1* | 0.111 | 0.021 | 7.58E-08 | 0.50 | 20 |
|  | ENSG00000074755 | 17 | *ZZEF1* | 0.181 | 0.039 | 2.73E-06 | 0.93 | 20 |

Supplementary Table 29. The gene expression of non-coding variants in 53 GTEx human tissues.

| **SNP Id** | **Gencode Id** | **Gene Symbol** | ***P*-Value** | **NES** | **Tissue** |
| --- | --- | --- | --- | --- | --- |
| rs13188193 | ENSG00000145730.20 | *PAM* | 1.00E-07 | 0.45 | Whole Blood |
| rs13188193 | ENSG00000175749.11 | *EIF3KP1* | 7.60E-07 | 0.61 | Artery - Tibial |
| rs13188193 | ENSG00000145725.19 | *PPIP5K2* | 0.000032 | -0.15 | Whole Blood |
| rs13188193 | ENSG00000226926.7 | *PDZPH1P* | 0.000068 | 0.56 | Skin - Not Sun Exposed (Suprapubic) |
| rs13188193 | ENSG00000226926.7 | *PDZPH1P* | 0.000082 | 0.53 | Esophagus - Muscularis |
| rs13188193 | ENSG00000175749.11 | *EIF3KP1* | 0.000084 | 0.67 | Breast - Mammary Tissue |
| rs13188193 | ENSG00000226926.7 | *PDZPH1P* | 0.00011 | 0.39 | Skin - Sun Exposed (Lower leg) |
| rs7531118 | ENSG00000227207.2 | *RPL31P12* | 3.00E-21 | -0.78 | Brain - Cerebellum |
| rs7531118 | ENSG00000227207.2 | *RPL31P12* | 5.30E-19 | -0.81 | Brain - Cerebellar Hemisphere |
| rs7531118 | ENSG00000172260.14 | *NEGR1* | 4.60E-07 | 0.18 | Brain - Putamen (basal ganglia) |
| rs7531118 | ENSG00000172260.14 | *NEGR1* | 7.70E-07 | 0.15 | Brain - Nucleus accumbens (basal ganglia) |
| rs7531118 | ENSG00000172260.14 | *NEGR1* | 0.000015 | 0.12 | Brain - Caudate (basal ganglia) |

Supplementary Table 30. S-PrediXcan TWAS data processing and multiple-testing summary.

| Trait | Input GWAS rows | Mapped model variants | Gene-tissue tests | FDR<0.05 gene-tissue associations | Bonferroni<0.05 gene-tissue associations |
| --- | --- | --- | --- | --- | --- |
| BMI | 2336269 | 21916 | 53265 | 11254 | 2419 |
| T2D | 5053015 | 43053 | 97192 | 3600 | 368 |

Supplementary Table 31. Tissue-level S-PrediXcan TWAS summary for BMI and T2D.

| Trait | Brain tissue | N genes tested | N FDR<0.05 | N Bonferroni<0.05 | N multi-SNP genes tested | N multi-SNP FDR<0.05 | Top gene | Top P | Top Z |
| --- | --- | --- | --- | --- | --- | --- | --- | --- | --- |
| BMI | Brain Amygdala | 3502 | 745 | 147 | 274 | 61 | MTCH2 | 7.50E-46 | 14.2 |
| BMI | Brain Anterior cingulate cortex BA24 | 3923 | 824 | 181 | 380 | 75 | MC4R | 4.70E-71 | -17.8 |
| BMI | Brain Caudate basal ganglia | 4365 | 919 | 205 | 451 | 103 | ADCY3 | 1.20E-54 | -15.6 |
| BMI | Brain Cerebellar Hemisphere | 4413 | 962 | 218 | 509 | 94 | NDUFS3 | 3.00E-50 | 14.9 |
| BMI | Brain Cerebellum | 4646 | 988 | 226 | 542 | 106 | NDUFS3 | 3.00E-50 | 14.9 |
| BMI | Brain Cortex | 4500 | 949 | 203 | 488 | 98 | ADCY3 | 8.20E-77 | -18.5 |
| BMI | Brain Frontal Cortex BA9 | 4312 | 907 | 210 | 441 | 88 | MC4R | 4.70E-71 | -17.8 |
| BMI | Brain Hippocampus | 3982 | 824 | 178 | 394 | 90 | MTCH2 | 7.50E-46 | 14.2 |
| BMI | Brain Hypothalamus | 3986 | 859 | 168 | 369 | 69 | MTCH2 | 7.50E-46 | 14.2 |
| BMI | Brain Nucleus accumbens basal ganglia | 4285 | 892 | 188 | 455 | 84 | RBM6 | 1.20E-41 | -13.5 |
| BMI | Brain Putamen basal ganglia | 4238 | 891 | 188 | 444 | 87 | RP11-1348G14.4 | 1.50E-58 | -16.1 |
| BMI | Brain Spinal cord cervical c-1 | 3695 | 757 | 158 | 296 | 53 | EFR3B | 2.70E-45 | -14.1 |
| BMI | Brain Substantia nigra | 3418 | 737 | 149 | 292 | 55 | RBM6 | 1.20E-41 | -13.5 |
| T2D | Brain Amygdala | 6778 | 250 | 23 | 1117 | 39 | LINC01126 | 5.36E-17 | -8.38 |
| T2D | Brain Anterior cingulate cortex BA24 | 7209 | 272 | 33 | 1455 | 41 | CTRB2 | 1.65E-17 | -8.52 |
| T2D | Brain Caudate basal ganglia | 7873 | 284 | 27 | 1759 | 56 | CTRB2 | 1.65E-17 | -8.52 |
| T2D | Brain Cerebellar Hemisphere | 7751 | 286 | 33 | 1799 | 56 | WFS1 | 2.36E-24 | 10.2 |
| T2D | Brain Cerebellum | 7970 | 283 | 30 | 1995 | 71 | WFS1 | 1.34E-28 | 11.1 |
| T2D | Brain Cortex | 8087 | 309 | 28 | 1862 | 60 | CDKN2B | 2.15E-16 | 8.21 |
| T2D | Brain Frontal Cortex BA9 | 7779 | 293 | 29 | 1653 | 64 | WFS1 | 3.98E-24 | 10.1 |
| T2D | Brain Hippocampus | 7338 | 265 | 26 | 1475 | 56 | CDKN2B | 2.15E-16 | 8.21 |
| T2D | Brain Hypothalamus | 7379 | 262 | 29 | 1476 | 60 | LINC01126 | 5.36E-17 | -8.38 |
| T2D | Brain Nucleus accumbens basal ganglia | 7812 | 300 | 26 | 1716 | 59 | ZFP36L2 | 5.36E-17 | 8.38 |
| T2D | Brain Putamen basal ganglia | 7642 | 294 | 31 | 1621 | 55 | WFS1 | 9.23E-24 | -10 |
| T2D | Brain Spinal cord cervical c-1 | 7014 | 276 | 33 | 1252 | 51 | LINC01126 | 5.36E-17 | -8.38 |
| T2D | Brain Substantia nigra | 6560 | 226 | 20 | 1143 | 42 | CTRB2 | 1.65E-17 | -8.52 |

Supplementary Table 32. Top multi-SNP S-PrediXcan genes by trait.

| Trait | Best tissue | Gene symbol | Ensembl gene | Z | P | FDR | N SNPs used | N SNPs in model |
| --- | --- | --- | --- | --- | --- | --- | --- | --- |
| BMI | Brain Hypothalamus | INO80E | ENSG00000169592.14 | -12.3 | 1.13E-34 | 8.14E-32 | 2 | 2 |
| BMI | Brain Cortex | NPC1 | ENSG00000141458.12 | -11.5 | 9.53E-31 | 4.03E-28 | 2 | 4 |
| BMI | Brain Frontal Cortex BA9 | NPC1 | ENSG00000141458.12 | -11.5 | 1.32E-30 | 5.56E-28 | 2 | 2 |
| BMI | Brain Hypothalamus | ZNF668 | ENSG00000167394.12 | -11.3 | 1.02E-29 | 3.95E-27 | 2 | 2 |
| BMI | Brain Amygdala | HSD17B12 | ENSG00000149084.12 | -11 | 5.47E-28 | 2.08E-25 | 2 | 2 |
| BMI | Brain Cerebellar Hemisphere | POLK | ENSG00000122008.15 | -11 | 6.14E-28 | 2.29E-25 | 2 | 2 |
| BMI | Brain Caudate basal ganglia | MAP2K5 | ENSG00000137764.19 | 10.9 | 1.60E-27 | 5.88E-25 | 2 | 3 |
| BMI | Brain Cerebellar Hemisphere | TBX6 | ENSG00000149922.10 | 10.8 | 5.49E-27 | 1.99E-24 | 2 | 4 |
| BMI | Brain Cerebellum | MAP2K5 | ENSG00000137764.19 | 10.7 | 6.40E-27 | 2.30E-24 | 2 | 2 |
| BMI | Brain Caudate basal ganglia | ZNF646 | ENSG00000167395.10 | 10.4 | 2.89E-25 | 9.94E-23 | 2 | 2 |
| BMI | Brain Cortex | CTC-366B18.4 | ENSG00000272040.1 | 9.61 | 7.52E-22 | 2.01E-19 | 2 | 3 |
| BMI | Brain Hippocampus | LINC00461 | ENSG00000245526.10 | -9.46 | 3.00E-21 | 7.17E-19 | 2 | 2 |
| BMI | Brain Anterior cingulate cortex BA24 | CTC-498M16.4 | ENSG00000271904.1 | -8.81 | 1.28E-18 | 2.52E-16 | 2 | 2 |
| BMI | Brain Cerebellum | NLRC3 | ENSG00000167984.17 | -8.49 | 2.13E-17 | 4.03E-15 | 2 | 4 |
| BMI | Brain Nucleus accumbens basal ganglia | DHX36 | ENSG00000174953.13 | -8.25 | 1.61E-16 | 2.72E-14 | 2 | 2 |
| BMI | Brain Putamen basal ganglia | CDK5RAP3 | ENSG00000108465.14 | -8.23 | 1.87E-16 | 3.16E-14 | 2 | 3 |
| BMI | Brain Anterior cingulate cortex BA24 | MARK3 | ENSG00000075413.17 | -8.14 | 3.94E-16 | 6.38E-14 | 2 | 3 |
| BMI | Brain Hippocampus | DHX36 | ENSG00000174953.13 | -7.98 | 1.52E-15 | 2.20E-13 | 2 | 2 |
| BMI | Brain Caudate basal ganglia | LINCR-0001 | ENSG00000253641.5 | 7.97 | 1.63E-15 | 2.35E-13 | 2 | 3 |
| BMI | Brain Nucleus accumbens basal ganglia | NIM1K | ENSG00000177453.7 | -7.92 | 2.40E-15 | 3.37E-13 | 2 | 2 |
| BMI | Brain Hypothalamus | ATP5G1 | ENSG00000159199.13 | 7.91 | 2.58E-15 | 3.62E-13 | 2 | 3 |
| BMI | Brain Frontal Cortex BA9 | PIK3C3 | ENSG00000078142.12 | -7.86 | 3.87E-15 | 5.35E-13 | 2 | 2 |
| BMI | Brain Anterior cingulate cortex BA24 | PIK3C3 | ENSG00000078142.12 | -7.86 | 3.88E-15 | 5.35E-13 | 2 | 2 |
| BMI | Brain Cerebellar Hemisphere | PIK3C3 | ENSG00000078142.12 | -7.85 | 4.16E-15 | 5.61E-13 | 2 | 2 |
| BMI | Brain Anterior cingulate cortex BA24 | NLRC3 | ENSG00000167984.17 | -7.85 | 4.17E-15 | 5.61E-13 | 2 | 4 |
| T2D | Brain Cerebellum | WFS1 | ENSG00000109501.13 | 11.1 | 1.34E-28 | 1.30E-23 | 2 | 2 |
| T2D | Brain Hippocampus | NCR3LG1 | ENSG00000188211.8 | -8.07 | 6.98E-16 | 1.70E-12 | 2 | 2 |
| T2D | Brain Caudate basal ganglia | NCR3LG1 | ENSG00000188211.8 | -7.98 | 1.50E-15 | 3.56E-12 | 2 | 2 |
| T2D | Brain Nucleus accumbens basal ganglia | NUDT5 | ENSG00000165609.12 | -7.5 | 6.53E-14 | 1.30E-10 | 2 | 3 |
| T2D | Brain Spinal cord cervical c-1 | UBE2Z | ENSG00000159202.17 | 7.39 | 1.43E-13 | 2.79E-10 | 2 | 2 |
| T2D | Brain Hippocampus | UBE2Z | ENSG00000159202.17 | 7.37 | 1.72E-13 | 3.24E-10 | 2 | 2 |
| T2D | Brain Nucleus accumbens basal ganglia | UBE2Z | ENSG00000159202.17 | 7.36 | 1.81E-13 | 3.25E-10 | 2 | 2 |
| T2D | Brain Hypothalamus | ARAP1 | ENSG00000186635.14 | 7.33 | 2.32E-13 | 4.10E-10 | 2 | 2 |
| T2D | Brain Spinal cord cervical c-1 | SNF8 | ENSG00000159210.9 | 7.32 | 2.55E-13 | 4.43E-10 | 2 | 2 |
| T2D | Brain Frontal Cortex BA9 | KCNJ11 | ENSG00000187486.5 | 7.04 | 1.94E-12 | 3.00E-09 | 2 | 2 |
| T2D | Brain Cortex | UBE2Z | ENSG00000159202.17 | 6.83 | 8.44E-12 | 1.14E-08 | 2 | 2 |
| T2D | Brain Hypothalamus | KCNJ11 | ENSG00000187486.5 | 6.83 | 8.71E-12 | 1.16E-08 | 2 | 2 |
| T2D | Brain Caudate basal ganglia | ZZEF1 | ENSG00000074755.14 | 6.82 | 8.90E-12 | 1.17E-08 | 2 | 2 |
| T2D | Brain Cerebellar Hemisphere | CTRB2 | ENSG00000168928.12 | -6.75 | 1.45E-11 | 1.80E-08 | 2 | 2 |
| T2D | Brain Hypothalamus | ZZEF1 | ENSG00000074755.14 | 6.47 | 9.99E-11 | 1.12E-07 | 2 | 2 |
| T2D | Brain Cerebellum | ZZEF1 | ENSG00000074755.14 | 6.42 | 1.39E-10 | 1.46E-07 | 2 | 2 |
| T2D | Brain Hippocampus | MICB | ENSG00000204516.9 | 6.39 | 1.70E-10 | 1.72E-07 | 2 | 2 |
| T2D | Brain Caudate basal ganglia | MICB | ENSG00000204516.9 | 6.34 | 2.30E-10 | 2.19E-07 | 2 | 4 |
| T2D | Brain Hypothalamus | MICB | ENSG00000204516.9 | 6.32 | 2.69E-10 | 2.37E-07 | 2 | 3 |
| T2D | Brain Putamen basal ganglia | MICB | ENSG00000204516.9 | 6.29 | 3.09E-10 | 2.61E-07 | 3 | 3 |
| T2D | Brain Spinal cord cervical c-1 | NCR3LG1 | ENSG00000188211.8 | -6.21 | 5.16E-10 | 4.11E-07 | 2 | 2 |
| T2D | Brain Amygdala | MANSC4 | ENSG00000205693.3 | 6.19 | 6.13E-10 | 4.81E-07 | 2 | 2 |
| T2D | Brain Cerebellum | MICB | ENSG00000204516.9 | 6.16 | 7.48E-10 | 5.64E-07 | 2 | 4 |
| T2D | Brain Cerebellum | ATP5G1 | ENSG00000159199.13 | 6.14 | 8.23E-10 | 6.15E-07 | 2 | 3 |
| T2D | Brain Spinal cord cervical c-1 | UBE2E2 | ENSG00000182247.9 | 6.14 | 8.35E-10 | 6.16E-07 | 2 | 2 |

Supplementary Table 33. Shared BMI/T2D FDR-significant S-PrediXcan genes supported by multi-SNP prediction models.

| Gene symbol | BMI best tissue | BMI P | BMI FDR | BMI Z | BMI N SNPs | T2D best tissue | T2D P | T2D FDR | T2D Z | T2D N SNPs |
| --- | --- | --- | --- | --- | --- | --- | --- | --- | --- | --- |
| NPC1 | Brain Cortex | 9.53E-31 | 4.03E-28 | -11.5 | 2 | Brain Cortex | 1.29E-06 | 2.73E-04 | -4.84 | 3 |
| HSD17B12 | Brain Amygdala | 5.47E-28 | 2.08E-25 | -11 | 2 | Brain Amygdala | 2.49E-06 | 4.56E-04 | -4.71 | 2 |
| MARK3 | Brain Anterior cingulate cortex BA24 | 3.94E-16 | 6.38E-14 | -8.14 | 2 | Brain Anterior cingulate cortex BA24 | 2.93E-05 | 0.0027 | -4.18 | 3 |
| ENHO | Brain Cerebellum | 2.55E-09 | 1.01E-07 | 5.96 | 2 | Brain Cerebellum | 6.00E-05 | 0.00469 | 4.01 | 2 |
| LINCR-0001 | Brain Caudate basal ganglia | 1.63E-15 | 2.35E-13 | 7.97 | 2 | Brain Caudate basal ganglia | 7.99E-05 | 0.0056 | 3.94 | 3 |
| TAP1 | Brain Cerebellum | 1.07E-04 | 0.00126 | -3.87 | 2 | Brain Cerebellum | 1.83E-08 | 9.87E-06 | -5.63 | 3 |
| ALS2CL | Brain Frontal Cortex BA9 | 1.32E-04 | 0.00149 | 3.82 | 2 | Brain Cerebellar Hemisphere | 1.26E-04 | 0.00771 | 3.84 | 2 |
| RAB29 | Brain Cerebellum | 5.51E-06 | 9.83E-05 | -4.54 | 2 | Brain Hypothalamus | 1.51E-04 | 0.00898 | -3.79 | 2 |
| MAP2K5 | Brain Caudate basal ganglia | 1.60E-27 | 5.88E-25 | 10.9 | 2 | Brain Putamen basal ganglia | 2.19E-04 | 0.0115 | 3.7 | 2 |
| CTC-366B18.4 | Brain Cortex | 7.52E-22 | 2.01E-19 | 9.61 | 2 | Brain Putamen basal ganglia | 4.02E-04 | 0.0177 | 3.54 | 2 |
| DHX36 | Brain Nucleus accumbens basal ganglia | 1.61E-16 | 2.72E-14 | -8.25 | 2 | Brain Frontal Cortex BA9 | 6.97E-04 | 0.0265 | -3.39 | 2 |
| KIF24 | Brain Frontal Cortex BA9 | 1.35E-05 | 2.16E-04 | 4.35 | 2 | Brain Frontal Cortex BA9 | 8.46E-04 | 0.0302 | 3.34 | 2 |
| NUDT2 | Brain Nucleus accumbens basal ganglia | 2.00E-05 | 3.02E-04 | -4.26 | 2 | Brain Cerebellar Hemisphere | 8.87E-04 | 0.0312 | -3.32 | 2 |
| C1GALT1 | Brain Frontal Cortex BA9 | 0.00316 | 0.0196 | 2.95 | 2 | Brain Caudate basal ganglia | 8.17E-04 | 0.0293 | 3.35 | 2 |
| BHMT | Brain Caudate basal ganglia | 0.00372 | 0.0223 | -2.9 | 2 | Brain Caudate basal ganglia | 8.00E-07 | 1.86E-04 | -4.94 | 2 |
| ZNF484 | Brain Cerebellar Hemisphere | 0.00626 | 0.0334 | -2.73 | 2 | Brain Cerebellar Hemisphere | 0.00148 | 0.0429 | 3.18 | 2 |
| TIMP4 | Brain Nucleus accumbens basal ganglia | 0.0082 | 0.041 | 2.64 | 2 | Brain Substantia nigra | 2.66E-07 | 8.19E-05 | 5.15 | 2 |
| LEPROT | Brain Putamen basal ganglia | 0.00857 | 0.0426 | -2.63 | 2 | Brain Cerebellum | 5.96E-04 | 0.0236 | 3.43 | 2 |
| MFSD4B | Brain Amygdala | 0.00985 | 0.0472 | 2.58 | 2 | Brain Amygdala | 2.87E-04 | 0.0139 | 3.63 | 2 |

Supplementary Table 34. Descriptive Enrichr gene-set screen for shared multi-SNP BMI/T2D TWAS genes.

| Library | Rank | Term | P | Adjusted P | Overlapping genes | N input genes |
| --- | --- | --- | --- | --- | --- | --- |
| GO_Biological_Process_2023 | 1 | Positive Regulation Of Macromolecule Metabolic Process (GO:0010604) | 0.00398 | 0.0672 | DHX36;RAB29;MAP2K5 | 18 |
| GO_Biological_Process_2023 | 2 | Regulation Of Growth Hormone Receptor Signaling Pathway (GO:0060398) | 0.00449 | 0.0672 | LEPROT | 18 |
| GO_Biological_Process_2023 | 3 | Peptide Transport (GO:0015833) | 0.00539 | 0.0672 | TAP1 | 18 |
| GO_Biological_Process_2023 | 4 | Protein Localization To Ciliary Membrane (GO:1903441) | 0.00539 | 0.0672 | RAB29 | 18 |
| GO_Biological_Process_2023 | 5 | G-quadruplex DNA Unwinding (GO:0044806) | 0.00539 | 0.0672 | DHX36 | 18 |
| KEGG_2021_Human | 1 | Fatty acid elongation | 0.024 | 0.122 | HSD17B12 | 18 |
| KEGG_2021_Human | 2 | Biosynthesis of unsaturated fatty acids | 0.024 | 0.122 | HSD17B12 | 18 |
| KEGG_2021_Human | 3 | Mucin type O-glycan biosynthesis | 0.0319 | 0.122 | C1GALT1 | 18 |
| KEGG_2021_Human | 4 | Primary immunodeficiency | 0.0337 | 0.122 | TAP1 | 18 |
| KEGG_2021_Human | 5 | Glycine, serine and threonine metabolism | 0.0354 | 0.122 | BHMT | 18 |
| Reactome_2022 | 1 | Choline Catabolism R-HSA-6798163 | 0.00539 | 0.126 | BHMT | 18 |
| Reactome_2022 | 2 | DEx/H-box Helicases Activate Type I IFN And Inflammatory Cytokines Production R-HSA-3134963 | 0.00628 | 0.126 | DHX36 | 18 |
| Reactome_2022 | 3 | Signaling By Leptin R-HSA-2586552 | 0.00986 | 0.126 | LEPROT | 18 |
| Reactome_2022 | 4 | Androgen Biosynthesis R-HSA-193048 | 0.00986 | 0.126 | HSD17B12 | 18 |
| Reactome_2022 | 5 | Defective C1GALT1C1 Causes TNPS R-HSA-5083632 | 0.017 | 0.126 | C1GALT1 | 18 |
